# Spontaneous Non‐Catalyzed Molecular Reactions and Interactions in the Human Body: Biomedical Implications

**DOI:** 10.1002/advs.202524277

**Published:** 2026-05-25

**Authors:** Yuhao Cai, Chao Zhao

**Affiliations:** ^1^ Department of Chemical and Biomolecular Engineering University of Massachusetts Amherst Amherst Massachusetts USA; ^2^ Department of Biomedical Engineering University of Massachusetts Amherst Amherst Massachusetts USA

**Keywords:** bioorthogonal ligation, boronate esterification, non‐catalyzed chemistry, schiff base formation, spontaneous reactions, thiol‐michael addition

## Abstract

The human body functions as a natural reactor for a vast network of chemical and biological reactions and physical interactions among small molecules, proteins, cells, and numerous other components. These reactions/interactions are essential for maintaining normal physiological functions. From an engineering perspective, these molecular reactions and interactions can be exploited for disease diagnosis, therapeutic interventions, and a variety of biomedical applications. In this review, we summarize non‐catalyzed molecular reactions and interactions that occur spontaneously under physiological conditions without enzymatic mediation. We discuss their biomedical implications in disease diagnosis, drug delivery, and biomaterials for regenerative and cellular engineering. Looking forward, we highlight emerging opportunities to leverage these spontaneous non‐catalyzed reactions and interactions in drug design, nonenzymatic activation of genome editing in synthetic biology, and non‐genetic surface modification and activation of immune cells.

## Abstract 2

The human body acts as a natural reactor where spontaneous molecular reactions and interactions support physiological function. This review summarizes non‐catalyzed processes that occur under physiological conditions without enzymatic mediation and discusses their biomedical applications in diagnosis, drug delivery, regenerative biomaterials, synthetic biology, drug design, and non‐genetic immune cell engineering.

**Abbreviations**
2APBA: 2‐acrylamidophenylboronic acid
4aPEG‐GLD: 4‐arm poly(ethylene glycol) bearing gluconic‐acid diol termini
ABPP: Activity‐based protein profiling
ABTS: 2,2’‐Azino‐bis(3‐ethylbenzothiazoline‐6‐sulfonic acid)
ADC: Antibody‐drug conjugate
AF: Auristatin F
AF488: Alexa Fluor 488
ALN: Alendronate
ASCT2: Alanine‐serine‐cysteine transporter 2
AUC: Area under the curve
BAP: Bioorthogonal activatable probe
BCN: Bicyclononyne
BNP: Bioadhesive nanoparticles
CA: Cholic acid
CA‐125: Cancer antigen 125
CB: Cucurbiturils
CCL151: CCL151 fibroblasts (cell line designation)
CD133: Cluster of differentiation 133
CD22: Cluster of differentiation 22
CD33: Cluster of differentiation 33
CD52: Cluster of differentiation 52
CD8: Cluster of differentiation 8
CDC: Cinnamaldehyde‐diethylenetriamine‐cinnamaldehyde dimer
CFU: Colony‐forming units
CKD: Chronic kidney disease
CMC: Critical micellization concentration
CRISPR/Cas: Clustered Regularly Interspaced Short Palindromic Repeats‐Associated Protein
CuAAC: Copper(I)‐catalyzed azide‐alkyne cycloaddition
DAR: Drug‐to‐antibody Ratio
DBCO: Dibenzocyclooctyne
DC: Dendritic cell
DiPBA: Diboronate
DMP: Dimethyl pimelimidate
DMS: Dimethyl suberimidate
DNA: Deoxyribonucleic acid
DOX: Doxorubicin
DOX‐hyd‐PEG‐ALN: Doxorubicin‐hydrazone‐poly(ethylene glycol)‐alendronate
DTBP: Dimethyl 3,3′‐dithiobispropionimidate
EB: Epothilone B
EDC: 1‐Ethyl‐3‐(3‐dimethylaminopropyl)carbodiimide
ELISA: Enzyme‐linked immunosorbent assay
EV: Extracellular vesicle
Fc: Ferrocene
FDA: Food and Drug Administration
FEDV: fluoroedavone
FPBA: 3‐fluoro‐phenylboronic acid
GSH: Glutathione
GTT: Glucose tolerance test
HA: Hyaluronic acid
H&E: Hematoxylin and eosin
HEK293: Human embryonic kidney 293
HER2: Human epidermal growth factor receptor 2
HIV‐1: Human immunodeficiency virus 1
HRP: Horseradish peroxidase
HS‐5: HS‐5 cells (stromal cell line designation)
IC50: Half‐maximal inhibitory concentration
ICD: Immunogenic cell death
iEDDA: Inverse electron‐demand Diels‐Alder reaction
IL: Interleukin
KRAS: Kirsten rat sarcoma viral oncogene homolog
KRAS^G12C^
: KRAS Glysine‐12 to Cysteine mutant
LOD: limit of detection
MDA‐MB‐231: MDA‐MB‐231 breast cancer cell line
MIC: Minimum inhibitory concentration
MMAE: Monomethyl auristatin E
MN: microneedle
MP: 6‐Mercaptopurine
NHS: N‐Hydroxysuccinimide
NIR: Near‐infrared
NNP: Non‐bioadhesive nanoparticles
PAGE: Poly(allyl glycidyl ether)
PBA: Phenylboronic acid
PBD: pyrrolobenzodiazepine
PBS: Phosphate‐buffered saline
PD‐1: Programmed Cell Death Protein 1
PDB: Pyrrolobenzodiazepine
PET: positron emission tomography
PD‐L1: Programmed cell death ligand 1
PEG: Poly(ethylene glycol)
PL‐A: Payload A
PLA: Polylactic Acid
PL‐B: Payload B
PLP: Pyridoxal 5’‐phosphate
PTA: cis‐3‐(9H‐purin‐6‐ylthio)acrylic acid
PTT: Photothermal therapy
RCS: Reactive carbonyl species
RNA: Ribonucleic acid
RNS: Reactive Nitrogen Species
ROS: Reactive Oxygen Species
SPAAC: Strain‐promoted azide‐alkyne cycloaddition
SRF: Sorafenib
SQP22: TCO‐modified MMAE derivative
TCO: Trans‐cyclooctene
TdLN: tumor‐draining lymph node
THF: tetrahydrofolate
TM4: TM4 cells, a well‐established, non‐tumorigenic mouse testicular Sertoli cell line
TTX: Tetrodotoxin
UV: Ultraviolet

## 1 Introduction

The human body functions as a natural chemical reactor, a highly dynamic biochemical network in which myriad reactions and interactions occur continuously to sustain homeostasis and life functions [1, 2, 3]. Broadly, these reactions and interactions can be categorized into two major modalities: (i) enzyme‐catalyzed processes, which dominate metabolic flux and provide significant rate acceleration and specificity [4, 5, 6, 7, 8, 9], and (ii) spontaneous, non‐catalyzed processes that occur in aqueous physiological environments due to the intrinsic reactivity of biomolecules and their assemblies. These processes include covalent bond formation and non‐covalent physical interactions (e.g., hydrophobic interactions, host‐guest recognition, etc.) [10, 11, 12, 13]. Both modes occur natively in vivo and can be purposefully harnessed for disease diagnostics, therapeutic interventions, and broader biomedical engineering applications [14, 15].

Almost all metabolic reactions are enzyme‐catalyzed processes. It is estimated that human cells collectively perform approximately 8000–13 000 distinct metabolic reactions [16]. Moreover, although many core metabolic pathways have been studied extensively and are well characterized, such as glycolysis [17, 18], citric acid cycle [19], pentose phosphate pathway [20, 21], a significant portion of metabolism is still not fully understood. For example, the enzymes involved, intermediate metabolites, and regulatory mechanisms remain unclear for many metabolic processes. Even for the metabolic reactions with well‐understood mechanisms, the overall reaction behavior can still be influenced by several factors, including enzyme expression level, tissue distribution, metabolic state, and susceptibility to enzyme degradation or inhibition [22, 23]. In contrast, the number of non‐catalyzed processes is much smaller, and their mechanisms are generally less complex because no enzymes are involved. As a result, these mechanisms are well understood and more controllable. Therefore, compared with enzyme‐catalyzed processes, non‐catalyzed reactions and interactions are typically more readily adaptable and easier to engineer for biomedical applications.

**FIGURE 1:**
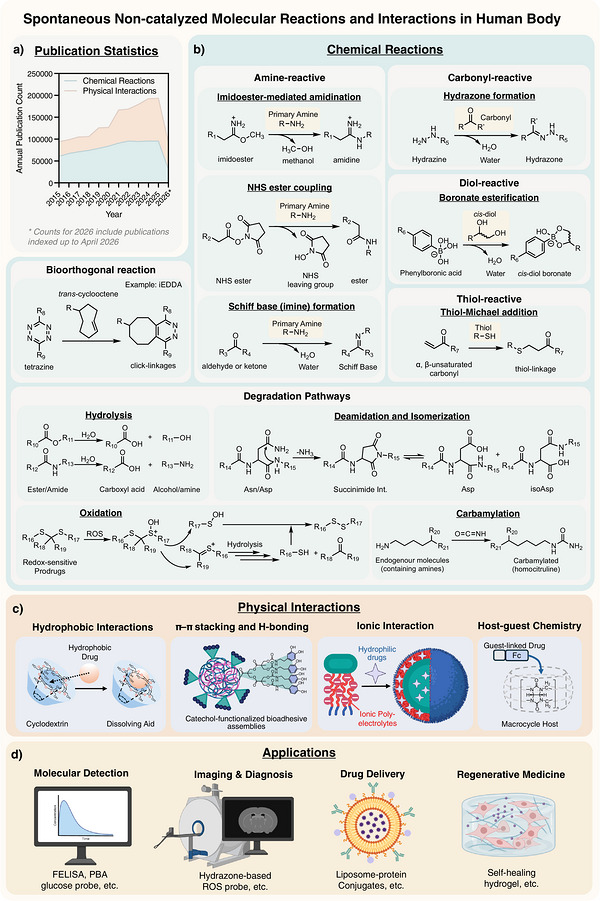
Spontaneous non‐catalyzed reactions and physical interactions operative in the human body and their biomedical applications. (a) Publication statistics of spontaneous non‐catalyzed reactions and physical interactions over the past decade. Counts for 2026 include publications indexed through April 2026. (b) Spontaneous non‐catalyzed reactions, including imidoester‐mediated amidination, NHS ester coupling, Schiff‐base (imine) formation, hydrazone formation, boronate esterification, thiol‐Michael addition, and bioorthogonal reactions, can proceed efficiently under physiological conditions and enable selective covalent modification of biomolecules. Representative degradation pathways are also shown, including hydrolysis, deamidation/isomerization, oxidation, and carbamylation. (c) Complementary non‐covalent mechanisms, including hydrophobic interactions, *π*–*π* stacking and hydrogen (H)‐bonding, ionic interactions, and host–guest chemistry, promote reversible molecular recognition, solubilization, and assembly in aqueous environments. (d) Together, these reactions and interactions underpin diverse biomedical applications, including molecular detection, imaging and diagnosis, drug delivery, and regenerative medicine.

Understanding spontaneous, non‐catalyzed processes is essential for elucidating in vivo reactivity. These processes show how biomolecules can form or transform chemical bonds within the mild aqueous environment of living systems, which provides fundamental insights into the chemistry underlying biological function [24, 25, 26]. This natural reactivity can be harnessed for therapeutic intervention [27, 28, 29, 30]. Spontaneous covalent coupling reactions have inspired targeted covalent inhibitors [27, 31, 32]. Related principles can enable “soft” drugs that predictably self‐inactivate after leaving the target site, thereby reducing systemic toxicity. They also support self‐regulated biomaterials that exploit inherent degradation or rearrangement kinetics for controlled drug release or tissue remodeling [33, 34, 35, 36, 37]. Moreover, enzyme‐free conjugation strategies are also emerging in immune engineering. Examples include in situ covalent programming of T cells and selective in vivo antibody modification to augment antitumor responses [12, 38]. This growing interest is also reflected in recent publication activity. Based on Google Scholar searches, reports related to the spontaneous non‐catalyzed reactions that can operative in the human body increased from approximately 60,177 in the year 2015 to 95,185 in the year 2025. Reports related to non‐covalent interactions that can operative in the human body increased from approximately 93,620 in the year 2015 to 193,000 in the year 2025 (Figure 1a). From January to April 2026, about 30,813 reports were published for chemical reactions, while 91,760 reports were published for non‐covalent interactions.

In this review, we summarize the current understanding of spontaneous, non‐catalyzed chemical reactions and non‐covalent physical interactions that are feasible within the human body (Figure 1b,c, Table 1). We discuss their mechanistic diversity, kinetic behavior, and biomedical potential (Figure 1d, Tables 2 and 3). The review specifically emphasizes spontaneous reactions and non‐covalent interactions that can be intentionally used for molecular assembly, bioconjugation, or biomedical function. Particular attention is given to amine‐reactive chemistry (e.g., imidoester‐mediated amidination) [39, 40, 41], carbonyl‐reactive processes (hydrazone formation) [42, 43], diol‐reactive boronate esterification [10], thiol‐reactive Michael additions [44, 45, 46], bioorthogonal chemistry [11, 47, 48, 49], and selected non‐covalent interactions (hydrophobic interactions, host–guest recognition, hydrogen bonding, *π*–*π* stacking, and ionic interactions) [50, 51]. We evaluate their biomedical implications in point‐of‐care diagnostics, site‐biased drug delivery, and smart dynamic biointerfaces and biomaterials for regenerative medicine. In addition, we briefly summarize a special group non‐catalyzed degradation pathways, such as cleavage‐driven processes (e.g., hydrolysis) and chemical damage processes (e.g., oxidation, deamidation, isomerization, carbamylation) [52, 53]. Looking forward, we highlight emerging opportunities to leverage this spontaneous non‐catalyzed chemistry repertoire in smart drug design (including self‐neutralizing “soft” therapeutics), nonenzymatic activation of genome editing within minimal‐component synthetic biology, and insitu non‐genetic surface modification of immune cells. Moreover, we also outline design rules and validation benchmarks to guide potential translation.

**TABLE 1:** Spontaneous non‐catalyzed reactions and interactions with related biomedical uses.

Reaction / Interaction	Representative biomedical uses	Development Stage	Ref
Imidoester‐mediated Amidination	ZnO nanospindle imidoester functionalization for antifungal therapy	Preclinical	[65]
	Traut's reagent thiolation in ADC assembly	In vitro	[71]
	DTBP‐stabilized BCP granules for bone regeneration	Preclinical	[79]
NHS ester coupling	NHS‐ester ABPP probe for proteome‐wide hotspot mapping	In vitro	[87]
	NHS‐functionalized hydrogel microspheres for rapid amine biosensing	In vitro	[88, 89]
	Site‐directed NHS antibody conjugation for homogeneous diagnostics	In vitro	[90]
	NHS silver halide fiber biosensor for Alzheimer's screening	In vitro	[91]
	Lysine‐directed NHS linker conjugation in ADCs	Approved	[97]
	NHS‐coupled HIV‐1 trimer liposomes as immunogen platforms	Preclinical	[99]
	Anti‐CD133 NHS‐functionalized AuNPs for 5‐FU colorectal targeting	Preclinical	[101]
	NHS‐polymer‐coated gelatin patches for hemostatic tissue sealing	Preclinical	[102]
	NHS‐coupled hydrogel adhesive for soft bioelectronics	Preclinical	[104]
Schiff base (imine) formation	Nanozyme‐coupled Schiff base biosensing for clenbuterol detection	In vitro	[118]
	Schiff base optical biosensor for CA‐125 ovarian cancer screening	In vitro	[119]
	Bioadhesive nanoparticle sunblock via Schiff base tissue adhesion	Preclinical	[120]
	Ciprofloxacin BNP‐hydrogel for biofilm‐retentive topical delivery	Preclinical	[121]
	Aldehyde‐terminated BNPs for intraperitoneal epothilone B delivery	Preclinical	[122]
	Schiff‐base‐crosslinked PEU hydrogels for pH‐responsive stability	In vitro	[126]
	Schiff base gelatin nanofibers for tissue‐engineering scaffolds	In vitro	[127]
Hydrazone formation	Peroxynitrite imaging with a hydrazone‐based lysosomal fluorescent probe	In vitro	[145]
	Hydrazone‐mediated CRISPR/Cas12a biosensing for P. aeruginosa	In vitro	[146]
	Formaldehyde imaging with a hydrazine‐based NIR probe	Preclinical	[147]
	Hydralazine‐mediated reactive carbonyl scavenging	Preclinical	[131]
	Hydrazone‐linked nanocarriers for pH‐responsive doxorubicin release	Preclinical	[149]
	DOX‐hyd‐PEG‐ALN micelles for acid‐triggered bone‐targeted delivery	Preclinical	[150]
	Trifunctional arylhydrazone linkers for sequential dual‐payload release	In vitro	[151]
	Hydrazone‐crosslinked PEG hydrogels for cartilage matrix deposition	In vitro	[152]
Boronate esterification	PBA hydrogel optical diffuser for wearable glucose sensing	Preclinical	[190]
	Gold nanoparticle colorimetric probe for norepinephrine detection	In vitro	[191]
	Boronic‐acid quantum dots for dopamine fluorescence sensing	In vitro	[192]
	Glucose‐responsive PBA‐Pluronic thermogels for insulin delivery	Preclinical	[196]
	DiPBA‐refined glucose‐responsive insulin depots	Preclinical	[197]
	HA‐DiPBA carrier for cis‐diol‐modified insulin delivery	Preclinical	[178]
	TTX‐PBA polymeric nanoparticles for prolonged local anesthesia	Preclinical	[198]
	2APBA‐PVA self‐healing hydrogels for 3D co‐culture	In vitro	[199]
	PBA‐functionalized chitosan/Bioglass scaffolds for bone engineering	In vitro	[195]
	Boronate‐assisted split RNA assembly for synthetic biology	In vitro	[200]
Thiol‐Michael addition	KRAS^G12C^ covalent inhibition with acrylamide warheads	Approved^a^	[208]
	GSH‐activated PTA prodrug for 6‐MP release	Preclinical	[210]
	CDC dimersomes for GSH‐depleting sorafenib delivery	Preclinical	[211]
	Maleimide liposomes for GSH‐depletion‐assisted immunotherapy	Preclinical	[212]
	Reinforced granular hydrogels for skin regeneration via thiol‐Michael addition	Preclinical	[213]
	Thermoswitchable adhesives via thiol/vinyl ether Michael addition	In vitro	[214]
Bioorthogonal reaction	PD‐L1 imaging with an iEDDA‐activatable NIR probe	Preclinical	[244]
	ASCT2 glutamine‐uptake tracking with a bioorthogonal probe	Preclinical	[245]
	Site‐specific ADCs via genetically encoded azide click conjugation	Preclinical	[246]
	Chemoenzymatic tyrosine‐click antibody conjugation	Preclinical	[247]
	iEDDA‐activated MMAE protodrugs for localized tumor therapy	Preclinical	[248]
	TdLN‐targeted rapamycin delivery via azide‐DBCO SPAAC	Preclinical	[249]
	DNA‐peptide hydrogels for peripheral nerve regeneration via SPAAC	Preclinical	[250]
	EV‐crosslinked stress‐relaxing hydrogels via SPAAC	In vitro	[251, 252]
Hydrophobic interactions	β‐CD–meropenem inclusion complex for sustained antibacterial delivery	In vitro	[267]
π–π stacking and hydrogen bonding	π–π‐stabilized paclitaxel micelles for tumor regression	Preclinical	[293]
	Catechol hydrogel tapes for wet tissue adhesion and sensor fixation	Preclinical	[296]
Ionic interactions	Electrostatically loaded micelles for pH‐responsive doxorubicin delivery	In vitro	[301]
Host–guest chemistry	CB[7]‐hydrogel homing depot for site‐biased small‐molecule targeting	Preclinical	[314]
Hydrolysis	Timolol ester prodrug–hydrogel for hydrolysis‐controlled release	Preclinical	[340]
Deamidation and isomerization	Anti‐CD52 antibody deamidation causing 400‐fold affinity loss	In vitro	[351]
Oxidation	[^18^F]FEDV PET probe for in vivo oxidative‐stress imaging	Preclinical	[368]
Carbamylation	Carbamylated albumin as a mortality‐linked biomarker in CKD	Clinical	[370]

^a^ the specific inhibitor reported in the cited study was not FDA‐approved but the same acrylamide‐warhead strategy is used in FDA‐approved KRAS G12C inhibitors, including sotorasib (Lumakras) [406] and adagrasib (Krazati) [407]

**TABLE 2:** Representative endogenous and synthetic counterparts of spontaneous non‐catalyzed molecular reactions and interactions.

Reaction / Interaction^a^	In Vivo Reactants	In Vitro / Biomedical Counterparts	Ref
Imidoester‐mediated amidination	Lysine residues, protein N‐termini, amine groups on cell‐surface proteins	Imidoester‐functionalized probes, nanocarriers, and granules.	[65]
NHS ester coupling		NHS‐ester probe, nanocarriers.	[101]
		NHS‐ester polymer coated patches.	[102]
Schiff base (imine) formation		Carbonyl‐bearing Probes.	[119]
		Bioadhesive nanoparticles.	[122]
Hydrazone formation	Aldehydes/ketones on endogenous saccharides and glycoconjugates (glycoprotein, proteoglycan, etc.), and RCS (oxidative stress)	Clinically applied RCS scavengers.	[131]
		Hydrazine‐bearing probe.	[147]
		Hydrazone‐based pH‐responsive carriers.	[149]
		Hydrazone‐based dual payload carriers.	[151]
Boronate esterification	Glucose and other saccharides, nucleic acids, glycoproteins, catecholamines (e.g., dopamine, epinephrine, etc.)	PBA‐functionalized biosensors.	[192]
		PBA‐based glucose‐responsive hydrogels.	[196]
		PBA‐functionalized nanocarriers.	[198]
		Boronated nucleic acid fragments.	[200]
Thiol‐Michael addition	Cysteine, GSH, *N*‐Acetyl‐Cysteine, Co‐enzyme A	Protein cysteine residue covalent inhibitors.	[208]
		GSH‐activated prodrugs.	[210]
		GSH‐depleting agents.	[211]
Bioorthogonal reactions	Non‐natural bioorthogonal handlesb) pre‐installation is required.	Tetrazine‐bearing probes.	[244]
	Usually tetrazine‐ or TCO‐bearing antibody or biopolymers.	TCO‐bearing prodrugs or protodrugs.	[248]
Hydrophobic interactions	Lipid membranes	Liposomes, micelles, lipid nanoparticles	[273]
	Protein hydrophobic disks (e.g. albumin)	Fatty acylated proteins or peptides	[274]
Hydrogen bonding and/or π–π stacking	Protein–ligand, base pairs, aromatic residues	Catechol adhesives	[293]
		Aromatic nanocarriers	[296]
Ionic interactions	Electrostatic protein–DNA	Polyelectrolyte complexes, coacervates	[301]
Host–guest chemistry	Molecular chaperones, macrocycle hosts (cyclodextrins, cucurbiturils, calixarenes, pre‐installation is required)b)	ferrocene derivatives, adamantane, azobenzene, doxorubicin, curcumin, and peptides	[314]

^a)^ Degradation pathways are generally intramolecular processes, except for deteriorative carbamylation. Because they do not typically have corresponding in vivo/in vitro roles as intentional biomedical design strategies, they are not listed in Table 1.

^b)^ In the context of bioorthogonal reactions and Host‐guest chemistry, “In vivo reactants” here typically refer to pre‐installed synthetic handles introduced prior to dosing (e.g., by metabolic labeling or conjugation) and therefore present in vivo at the time of reaction, though not native to unmodified tissues.

**TABLE 3:** Kinetics‐based classification of non‐catalyzed strategies, advantages and disadvantages in biomedical context.

Kinetics class	Reaction / Interaction	Condition	Reversibility	Rate constants (k_2_, 37°C, pH 7.4)	Ref
Ultrafast (s–min)	Bioorthogonal Reactions	pH 7.4, PBS, 37°C	Irreversible (most); fast and selective	Staudinger: 10^−3^ m ^−1^·s^−1^ SPAAC: 10^−2^–10^2^ m ^−1^·s^−1^ iEDDA: 10^3^–10^6^ m ^−1^·s^−1^	[48]
	Thiol–Michael addition	pH 7–8, aqueous medium or PBS, 25°C–37°C	Mostly irreversible (some retro‐reactions)	10–10^4^ m ^−1^·s^−1^	[203]
	Boronate esterification	pH 7.4, PBS, 37°C	Reversible	1–50 m ^−1^·s^−1^ (unmodified PBA);	[183]
				10^2^–10^3^ m ^−1^·s^−1^ (engineered PBAs)	[185]
	Hydrophobic assembly	Physiological ionic strength (∼150 mm NaCl, pH 7.4, PBS), 37°C	Reversible	10^6^–10^10^ m ^−1^·s^−1^ diffusion‐limited	[378]
	*π*–*π* stacking + H‐bonding		Reversible	10^8^–10^10^ m ^−1^·s^−1^, diffusion‐limited	[379]
	Ionic interaction		Reversible	10^9^ m ^−1^·s^−1^, diffusion‐limited	[380]
	Host–guest chemistry		Reversible	10^7^–10^9^ m ^−1^·s^−1^, diffusion‐limited	[381]
Slow (10 min–h)	NHS ester coupling	pH 7.5–8.5, PBS, 25°C–37°C	Irreversible	10^−1^ m ^−1^·s^−1^	[385]
	Imidoester‐mediated amidination	pH 8–9, PBS, 25°C–37°C	Essentially irreversible	*k'* = 0.08–0.22 h^−1^ across pH ∼6.8–8.	[55]
	Schiff base (imine) formation	pH 7–8, aqueous buffers, 25°C–37°C	Reversible (reducible to stabilize)	up to 2.85 m ^−1^·min^−1^	[42]
	Hydrazone formation	pH 6–7.4, aqueous or aniline‐free buffers, 25°C–37°C	Reversible	2‐20 m ^−1^·s^−1^	[42]
Variable	Hydrolysis	pH ∼7.4, PBS/body fluids, 37°C	Mostly irreversible	Strongly bond‐dependent	[334]
	Deamidation and isomerization	pH ∼ 7.4, PBS/body fluids, 37°C	Reversible	Strongly dependent to pH, substance, conformation, etc.	[347]
	Oxidation	ROS/RNS‐containing pathological microenvironments, 37°C	Variable per conditions	Oxidant‐/substrate‐dependent	[359]
	Carbamylation	pH ∼ 7.4, PBS/body fluids, 37°C	Mostly irreversible	Strongly dependent cyanate burden and pathological progresses	[329]

## 2 Spontaneous Covalent Bond‐Forming Reactions

### 2.1 Amine‐Reactive Chemistry

**FIGURE 2:**
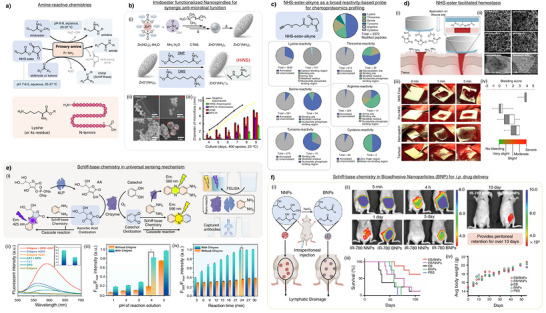
Overview of amine‐reactive chemistry and representative biomedical applications. (a). Overall formula of amine‐reactive chemistry, including imidoester‐mediated amidination, NHS ester coupling, and the formation of Schiff base (imine). Typical in vivo amine‐bearing molecules are lysine residues and N‐termini of proteins. (b). Imidoester functionalized zinc oxidized nanospindles for synergic antimicrobial therapy (i). DMP‐ or DMS‐modified nanospindles interact with bacteria and fungus (ii), achieving antibiotic cofunction with itraconazole (iii). DMP: dimethyl pimelimidate. DMS: dimethyl suberimidate. Adapted with permission. [65] Copyright 2021, American Chemical Society. (c). NHS‐ester‐alkyne used in chemoproteomics profiling of nucleophilic residues, including lysine, threonine, serine, arginine, tyrosine, and cysteine. Adapted with permission. [87] Copyright 2017, American Chemical Society. (d). Hemostatic gelatin coated with NHS‐functionalized poly(2‐oxazoline) (Pox‐NHS, i‐ii). NHS ester coupling forms covalent crosslinks among gelatin, blood protein, and tissue amines, creating a wound sealing gel that reduces bleeding (iii, iv). Adapted with permission. [102] Copyright 2017, American Chemical Society, distributed under CC BY License. (e). Schiff‐base chemistry‐coupled with a catechol oxidase‐like nanozyme (CHzyme) reaction for multi‐signal fluorescent sensing (i). The cascade enables ultra‐sensitive detection (ii, iii) and instant fluorescent emission (iv). Adapted with permission. [118] Copyright 2023 American Chemical Society. (f) Aldehyde‐functionalized bioadhesive nanoparticles (BNPs) adhere to abdominal inner wall via the formation of Schiff base, thereby prolong the local retention (ii) and sustained release of epothilone B (EB). This resulted in higher survival rate (iii) and sustained weight gaining (iv) than plain EB or non‐adhesive nanoparticles (NNPs) formulations. Adapted for noncommercial use under PNAS policy.  [122] Copyright 2016, National Academy of Sciences.

In amine‐reactive chemistry, primary or secondary amines function as nucleophiles due to the presence of a lone pair of electrons on the nitrogen atom. This lone pair can attack an electrophilic center on a reagent, leading to the formation of a covalent bond. Amine‐reactive chemistry is commonly employed to modify biomolecular primary amines, such as lysine ε‐amines and N‐terminal α‐amines, under aqueous, near‐physiological conditions, enabling robust protein/peptide bioconjugation [54, 55, 56]. There are many types of amine‐reactive chemistries, depending on the electrophilic functional group that reacts with the amine. In bioconjugation, the most common three categories are: (i) imidoester‐mediated amidination, providing stable cationic linkages [57, 58]; (ii) N‐hydroxysuccinimide (NHS) ester coupling, which enables rapid and efficient acylation of amines [59, 60], (iii) Schiff base (imine) formation between amines and carbonyl compounds (aldehyde or ketone), offering reversible conjugation under mild aqueous conditions (Figure 2a) [61]. Each reaction type offers unique advantages and applications, contributing significantly to advancements in bioconjugation and therapeutic development [62, 63, 64].

#### 2.1.1 Imidoester‐Mediated Amidination

Imidoester‐mediated amidination involves imidoesters serving as efficient amidinating reagents. These reagents react selectively with primary amines to generate stable amidine linkages (Figure 2a, Table 1) [65, 66]. This reaction occurs optimally in neutral to mildly basic buffer. The highest reaction efficiency is typically observed at pH 8–9, where amine nucleophilicity is enhanced by deprotonation. In biological systems, relevant nucleophilic partners include lysine ε‐amines, N‐terminal α‐amines, and other accessible primary amines on proteins, peptides, or amine‐bearing biomaterials (Table 2). Kinetically, imidoester‐mediated amidination shows apparent rate constants of *k’* = 0.08–0.22 h^−1^ over approximately pH 6.8‐8.0 (Table 3) [55]. Mechanistically, the amine attacks the electrophilic carbonyl carbon of the imidoester to form a tetrahedral intermediate, which collapses with release of an alkoxide, yielding an amidine (Figure 2a). At physiological pH, the resulting amidine is protonated, giving it a positive charge that can improve aqueous solubility and conjugate stability in biological environments.

Imidoester‐mediated amidination has been widely used for ex vivo analytical and preparative purposes. In enzyme immobilization, imidoesters can form stable amidine linkages with N‐terminus and lysine residues. Homobifunctional reagents such as dimethyl adipimidate, dimethyl pimelimidate (DMP), and dimethyl suberimidate (DMS) are therefore routinely employed to crosslink proteins or anchor them to carrier surfaces. This crosslinking can enhance conformational rigidity and thermal stability, thereby support biocatalyst reuse [67]. In proteomic and structural analyses, imidoester‐mediated amidination has been applied to selectively label accessible amines or probe subunit interfaces. These applications allow investigators to extract structural or interaction information while preserving the cationic character of modified sites [66, 68, 69].

In drug delivery systems, imidoester‐mediated amidination is mainly used to couple payloads or targeting components to antibodies and carrier platforms through primary‐amine‐directed reactions. A particularly relevant application is antibody‐drug conjugate (ADC) construction. In this context, 2‐iminothiolane (Traut's reagent) is the most widely used tool to amidinate lysine ε‐amines or N‐terminal α‐amines while introducing thiol handles, which can then react with maleimide‐bearing payloads through thiol‐Michael addition [70, 71]. More broadly, delivery‐oriented ligation strategies for liposomal nanocarriers and related liposome conjugates highlight the importance of imidoester‐mediated amidination. This chemistry can install targeting or functional components on carrier surfaces [72, 73]. A representative example is the work of Liu et al. [65]. They prepared homobifunctional imidoester‐coated zinc oxide nanospindles (HINS) by simply mixing nanospindle ZnO with DMP or DMS in aqueous suspension (Figure 2b). The nanospindle ZnO precursor carried a positively charged, amine‐containing surface. DMP or DMS was proposed to react with these surface amines during modification. The resulting HINS showed higher surface charge, better solubility, and improved biocompatibility. They also displayed enhanced antimicrobial efficacy against bacteria and fungi. In combination with itraconazole, HINS presented a synergic antifungal effect, achieving >90% fungal destruction at low dosage without detectable cytotoxicity or coagulation in both in vitro and in vivo studies. Similar functionalization principles have also been discussed for cationic polymers and silica‐based systems designed for targeted delivery [74, 75]. Taken together, these examples show that imidoester‐mediated amidination has been used not only for assembling antibody‐ and liposome‐based delivery constructs, but also for engineering functional therapeutic materials.

Imidoester‐mediated amidination has also been applied in regenerative medicine primarily to stabilize collagenous and gelatinous biomaterials for implantable or bone‐regenerative constructs. Early work showed that fibrous collagen crosslinked by DMS in 0.2 m Tris buffer (pH = 9) could be implemented for dermal implant, although residual DMS cytotoxicity required careful control [76]. Subsequent comparative studies further indicated that imidoester‐mediated amidination is able to modulate the physical properties and biocompatibility of collagen membranes relative to glutaraldehyde‐based systems [77, 78]. A representative example is the work of Kim et al. [79], who stabilized collagen‐ and gelatin‐coated porous biphasic calcium phosphate granules by dimethyl 3,3′‐dithiobispropionimidate (DTBP) crosslinking.

The broader biomedical (especially in vivo) translation of imidoester‐mediated amidination is constrained by several practical limitations. Imidoesters can undergo rapid hydrolysis in water which shortens their half‐life and reduces effective reactivity at physiological conditions [80]. In addition, cytotoxicity has been reported for some imidoesters at concentrations used for crosslinking (e.g., DMS in collagen crosslinking) [76]. This necessitates extensive washing and post‐treatment to mitigate residual toxicity for in vitro prepared agents while burdening the in vivo occurrence of this reaction. Their utility can also depend on specific substrate or material system, which may restrict performance across different biomedical context [81]. Together, these considerations underscore that while imidoester‐mediated amidination is a robust strategy for many biomedical applications, the in vivo utility of imidoester‐mediated amidination often requires careful reagent choice, dosing, and formulation/processing strategies to balance reactivity with stability and safety.

#### 2.1.2 NHS Ester Coupling

NHS ester coupling is an amine acylation in which a primary amine, typically the ε‐amino group of lysine or an N‐terminal amine, attacks an NHS‐activated carboxylic ester to form amide bonds under mild, typically slightly basic conditions (commonly pH 7.2–8.5) [40, 82]. Mechanistically, the reaction proceeds by nucleophilic acyl substitution, with NHS or sulfo‐NHS expelled as the leaving group (Figure 2a). In practice, coupling efficiency is governed by competition between productive aminolysis (reaction with amines) and hydrolysis (reaction with water) of the activated ester, so conversion depends strongly on pH, buffer composition, and ester structure [82]. In biological systems, lysine side chains, N‐terminal α‐amines, and other accessible primary amines on proteins, peptides, or amine‐bearing materials (Table 2) can participate in this chemistry. Kinetically, NHS esters react with amines with second‐order rate constants on the order of *k*_2_ = 10^−1^ m ^−1^ s^−1^ (Table 3).

NHS ester coupling has been widely adopted in in vitro analytical and diagnostic applications. Beyond routine attachment of fluorophores, affinity tags, drugs, and polymers to proteins and peptides [68, 83, 84, 85, 86]. NHS ester coupling is also adapted for chemoproteomics interrogation of protein activity. For instance, Ward et al. [87] used an NHS‐ester‐based probe to map ligandable nucleophilic hotspots via Activity‐based protein profiling (ABPP) across proteomes (Figure 2c). Using NHS‐ester‐alkyne probe, this research identified 3372 probe‐modified peptides. Nearly half of these sites (1639) were lysines, but the probe also captured serines (591), threonines (614), tyrosines (275), arginines (224), and a smaller number of cysteines (29). It also showed that NHS‐ester fragments could be tuned to engage specific lysine hotspots on proteins such as dihydropyrimidine dehydrogenase, aldehyde dehydrogenase 2, and glutathione S‐transferase theta 1, highlighting their value for covalent ligand discovery. Liu et al. [88] developed a universal amine‐capturing microparticle platform via NHS‐functionalized porous poly(acrylamide‐co‐acrylic acid) hydrogel microsphere. In this in vitro study, the NHS groups on the microspheres were used to capture primary amine‐bearing molecules and proteins. The investigators showed that the model protein R‐phycoerythrin fully penetrated the microspheres within 30 min and completed conjugation within 2 h. NHS‐functionalized silver halide fibers have also been developed as infrared biosensing platforms [91]. In this strategy, NHS esters were anchored onto the silver‐containing fiber surface and formed a chemisorbed monolayer. These exposed NHS ester groups were confirmed to immobilize bovine serum albumin (BSA) after a 1‐h incubation, as evidenced by the appearance of protein amide I/II bands and the concurrent loss of NHS ester features in the IR spectra. This approach thereby supports analysis of disease‐relevant targets in blood plasma or cerebrospinal fluid and has been proposed for miniaturized devices for Alzheimer's disease screening.

In the context of drug delivery and therapeutic applications, NHS ester coupling is utilized for payload installation, linker attachment, and carrier‐surface functionalization. A particularly essential role of NHS ester coupling is in ADC constructions, where NHS‐bearing heterobifunctional linkers acylate lysine residues on antibodies and thereby provides attachment of cytotoxic payloads for targeted cancer therapy [92, 93, 94, 95], as exemplified by Food and Drug Administration (FDA)‐approved ADCs Mylotarg (for cluster of differentiation [CD]33‐postive acute myeloid leukemia) [96]. Kadcyla (for human epidermal growth factor receptor 2 [HER2]‐positive breast cancer) [97], and Besponsa (for CD22‐postive B‐cell precursor acute lymphoblastic leukemia) [98]. NHS ester coupling has also been applied to liposome‐based therapeutic constructs. A clear exemplar is that Suleiman et al. [99], who used EDC/sulfo‐NHS chemistry to conjugate native‐like human immunodeficiency virus type 1 (HIV‐1) envelope (Env) trimers onto liposome surfaces. Sulfo‐NHS ester groups on the liposomes reacted with primary amines on Env trimers to form covalent amide bonds. The resulting liposome construct serves as a protein‐displaying immunogen platform to activate immune response rather than conventional payload delivery. Under optimized conditions, the system achieved about 40%–60% conjugation efficiency while preserving substantial trimer antigenicity, as indicated by ≥70% reactivity toward the HIV‐1 broadly neutralizing antibody PGT145 [99]. NHS ester coupling has also been extended to other nanoparticle (NP) functionalization [100]. In one such case, NHS ester coupling was used to conjugate polyethylene glycol (PEG)‐modified gold nanoparticles (PEG‐AuNPs) with anti‐CD133 monoclonal antibody, yielding PEG‐AuNPs‐CD133. 5‐fluorouracil (5‐FU) was then loaded noncovalently via the electron cloud of p back‐bonded carbonyl oxygen of PEG‐AuNPs. In an in vitro model of HCT116 colorectal cancer cells, which express CD133 at 92.4 ± 1.3%, 5‐FU‐PEG‐AuNPs‐CD133 reduced cell viability significantly more than 5‐FU‐PEG‐AuNPs without antibody conjugation, supporting that antibody conjugation promoted specific intracellular uptake [101].

In regenerative medicine, NHS ester coupling supports strengthened adhesion between biomaterials and tissue through covalent amide‐bond formation with accessible amines. For example, Boerman et al. [102] coated porous gelatin sponge patches with NHS‐functionalized poly(2‐oxazoline)s (Pox‐NHS, Figure 2d). The coating enabled NHS ester coupling with amine‐containing components at the wound site, including blood proteins, endogenous gelatin, and tissue. The materials retained porous sponge morphology, which supported blood absorption while preserving patch integrity. The patches were evaluated in vivo in heparinized pigs with liver and spleen injury models. In both models, Pox‐NHS‐coated patches showed favorable bleeding scores and effective hemostasis, overperforming commercially available controls Hemopatch and Tachosil. This increased adhesion and durability were beneficial because they help maintain patch‐wound contact, reduce leakage under wet conditions, and improve the reliability of intraoperative sealing [103]. More recently, Tian et al. [104] incorporated NHS‐ester coupling into a soft‐bioelectronic adhesive based on a sodium alginate‐polyacrylamide‐acrylic acid NHS hydrogel (SPAN) with a liquid chitosan adhesive layer. NHS ester groups in the SPAN hydrogel reacted with amine‐bearing tissue components, while the liquid chitosan layer further reinforced interfacial coupling and hydrogel cohesion. In rat subcutaneous muscle, these adhesive electrodes remained stably integrated for 35 days and supported long‐term electrophysiological recording with high signal‐to‐noise ratios. These studies collectively show that NHS ester coupling is valuable not only for immediate wound sealing, but also for maintaining durable material‐tissue integration in dynamic biological environments.

The broader use of NHS ester coupling in complex biological settings is limited by competing hydrolysis, incomplete site selectivity, and condition‐dependent conjugation efficiency. Under aqueous conditions, NHS esters can hydrolyze before productive aminolysis occurs, which shortens reagent lifetime and reduce coupling efficiency [105]. Furthermore, because multiple accessible amines are often present on proteins and material surfaces, NHS‐mediated modification can generate heterogeneous products with limited positional control [106] These issues are especially relevant in analytical and therapeutic systems that require controlled stoichiometry or reproducible surface modifications [60, 107]. Despite these challenges, the existing applications highlight the enduring significance of NHS esters in advancing scientific research and developing innovative therapeutic and diagnostic technologies.

#### 2.1.3 Schiff Base (Imine) Formation

Schiff base (imine, R‐CH = NR’) formation is a condensation reaction in which a primary amine reacts with an aldehyde or ketone to form a carbon‐nitrogen double bond under mild conditions, typically near neutral to mildly basic conditions (pH 7–8, Figure 2a, Table 1) [108]. Mechanistically, the amine first adds to the carbonyl group to form a carbinolamine intermediate, which then dehydrates to generate the imine linkage. In biological systems, amine‐bearing species such as lysine side chains or N‐terminal amines (Table 2) can participate in this reaction. Schiff base linkages also occur as transient intermediates in enzymatic processes. A well‐known example is pyridoxal‐5’‐phosphate (PLP)‐dependent enzymes. The aldehyde group of PLP forms a reversible imine with an active‐site lysine residues or with the amino group of the substrate. Such intermediates are central to reactions such as transamination and decarboxylation [109]. Under physiological conditions, Schiff base linkages are often dynamic and reversible, and both their formation kinetics and stability depend strongly on substrate structure, pH, and local microenvironment [110]. Representative second‐order formation rates have been reported up to 2.85 m ^−1^ min^−1^ (Table 3), with equilibrium constants also varying widely depending on the aldehyde and amine structure [111, 112]. Both reaction rate and product's stability are tunable through substrate design. Aromatic aldehydes often improve imine formation and persistence [113]. Boron‐assisted‐systems can stabilize the product through iminoboronate formation [114, 115]. If a more stable linkage is required, the imine can be converted into a secondary amine by reductive amination [116, 117].

In diagnostic probes, Schiff base formation is mainly used to construct analyte‐responsive sensing systems. By varying the aldehyde and amine components, Schiff‐base probes can be designed to alter fluorescence or colorimetric behavior in response to specific biomolecules or ions [111]. A particularly clear example is the work of Li et al. [118], who coupled Schiff base chemistry with a catechol oxidase‐like nanozyme (CHzyme) to create a multi‐signal fluorescent sensing platform (Figure 2e). CHzyme first oxidized catechol into a carbonyl‐bearing intermediate. The intermediate then reacted with *o*‐phenylenediamine through Schiff base formation to generate a fluorescence. The resulting cascade supported sensing of catechol, ascorbic acid, and alkaline phosphatase. It performed best at neutral pH, where the catalytic signal was strong and the background remained low, and the optimal reaction time was 24 min. The platform was further integrated into a ratiometric fluorescent enzyme‐linked immunosorbant assay (ELISA) for clenbuterol detection achieving a 15‐fold increase in sensitivity related to conventional ELISA. In addition, Abou‐Omar et al. [119] developed a nano‐gold/sol‐gel optical biosensor that contained a preformed bis‐imine Schiff base ligand, 2,2’‐((1E,1’E)‐(1,2‐phenylenebisazanylylidene))bis(enthane‐1‐yl‐ylidene)diphenol. This ligand was first complexed with Au(III) and then incorporated into the sensor film. The Schiff‐base‐bearing film emitted fluorescence at 423 nm, and cancer antigen‐125 (CA‐125) was quantified through quenching the signal. The biosensor showed a linear range of 2.0–127.9 U mL^−1^ and a detection limit of 1.45 U mL^−1^. These studies show that Schiff base formation can support diagnostic probe design not only as a linking reaction, but also as a chemically tunable process for biomarker recognition and optical signal alteration.

In drug delivery and therapeutic applications, Schiff base formation has been used to create bioadhesive nanoparticles (BNPs) that improve local retention and thereby enhance therapeutic efficacy. In these systems, aldehyde groups on NP surfaces form Schiff base linkages with primary amines on proteins in biological interfaces. This forms Schiff base linkages that increases adhesion to tissues or mucosal surfaces and thereby reduces premature clearance. For example, in a BNP‐based sunblock, encapsulation of padimate O in BNPs prevented epidermal penetration, increased ultraviolet (UV) absorbance by 20‐fold relative to free padimate O in water [120]. The formulation achieved anti‐UV protection comparable to commercial sunscreens with less than 5 wt.% of the UV‐filter concentration. It also significantly reduced double‐stranded deoxyribonucleic acid (DNA) breaks [120]. In a similar manner, a BNP‐hydrogel hybrid loaded with ciprofloxacin showed superior adhesion and antibiotic retention under high shear stress on bacterial films, mammalian cell monolayers, and mouse skin [121]. The system inhibited *Escherichia coli* biofilm formation under flow and caused no observable skin toxicity during 7 days of topical treatment [121]. This Schiff base formation‐derived adhesion strategy was later extended to intraperitoneal drug delivery. Specifically, Deng et al. [122] used aldehyde‐terminated BNPs to deliver epothilone B (EB) intraperitoneally against chemotherapy‐resistant uterine serous carcinoma (Figure 2f). Compared with free EB and non‐bioadhesive nanoparticles (NNPs) carrying the same drug, the BNP formulation prolonged peritoneal retention for over 10 days. It also produced stronger antitumor activity, reduced systemic toxicity, and sustained body weight gain in mice bearing intraperitoneal xenografts. At a dose of 0.5 mg kg^−1^ EB, 60% of the animals treated with BNPs survived beyond 110 days, whereas only 10% survived in the free‐EB and NNPs groups. These studies show that Schiff base formation can be exploited as a practical adhesion strategy for localized therapy, prolonged retention, and improved treatment performance.

In the context of regenerative medicine, Schiff base formation is used to generate dynamic crosslinks between aldehyde‐ and amine‐ functionalized components for network formation under mild conditions [123, 124]. The mechanical properties of resulting networks strongly depend on the stability and reversibility of the imine linkage, as well as on crosslinking density and polymer composition [124, 125]. For example, Pappalardo et al. [126] showed that Schiff‐base‐crosslinked poly(ether urethane) hydrogels remained stable for up to 27 days under physiological conditions (37°C and pH 7.4). Under acidic or basic conditions, the hydrogels underwent pH‐dependent swelling or dissolution. These findings suggest that Schiff base networks can be tuned for different biological microenvironments and may be useful where local pH differs from normal tissue conditions. Jaiswal et al. [127] synthesized a 2‐nitrobenzyl‐gelatin Schiff base derivative and used it to fabricate electrospun gelatin nanofiber matrices for tissue engineering. Compared with plain gelatin nanofibers, the incorporation of Schiff base chemistry significantly increased the modulus from 9.08 ± 1.5 MPa to 15.62 ± 2.8 MPa. In this setting, Schiff base formation contributed not only to the stabilized structural integrity of the matrix, but also to enhanced biological properties, as shown by a 73% increase in cell attachment and proliferation [127].

The broader biomedical use of Schiff base formation is limited by competing hydrolysis, variable linkage stability, and the tradeoff between dynamic reversibility and long‐term structural durability. Recent studies continue to emphasize that Schiff base formation in water is intrinsically challenged by competing hydrolysis, and that efficient use under physiological conditions often requires additional chemical stabilization (e.g., reduction to secondary amines) [128]. For biomaterials, the dynamic Schiff‐base exchange that offers injectability and self‐healing can also weaken the long‐term mechanical integrity [129, 130]. Accordingly, successful biomedical implementation usually requires careful tuning of imine structure and network architecture to balance dynamic function with persistence.

### 2.2 Hydrazone Formation (Carbonyl‐Reactive)

**FIGURE 3:**
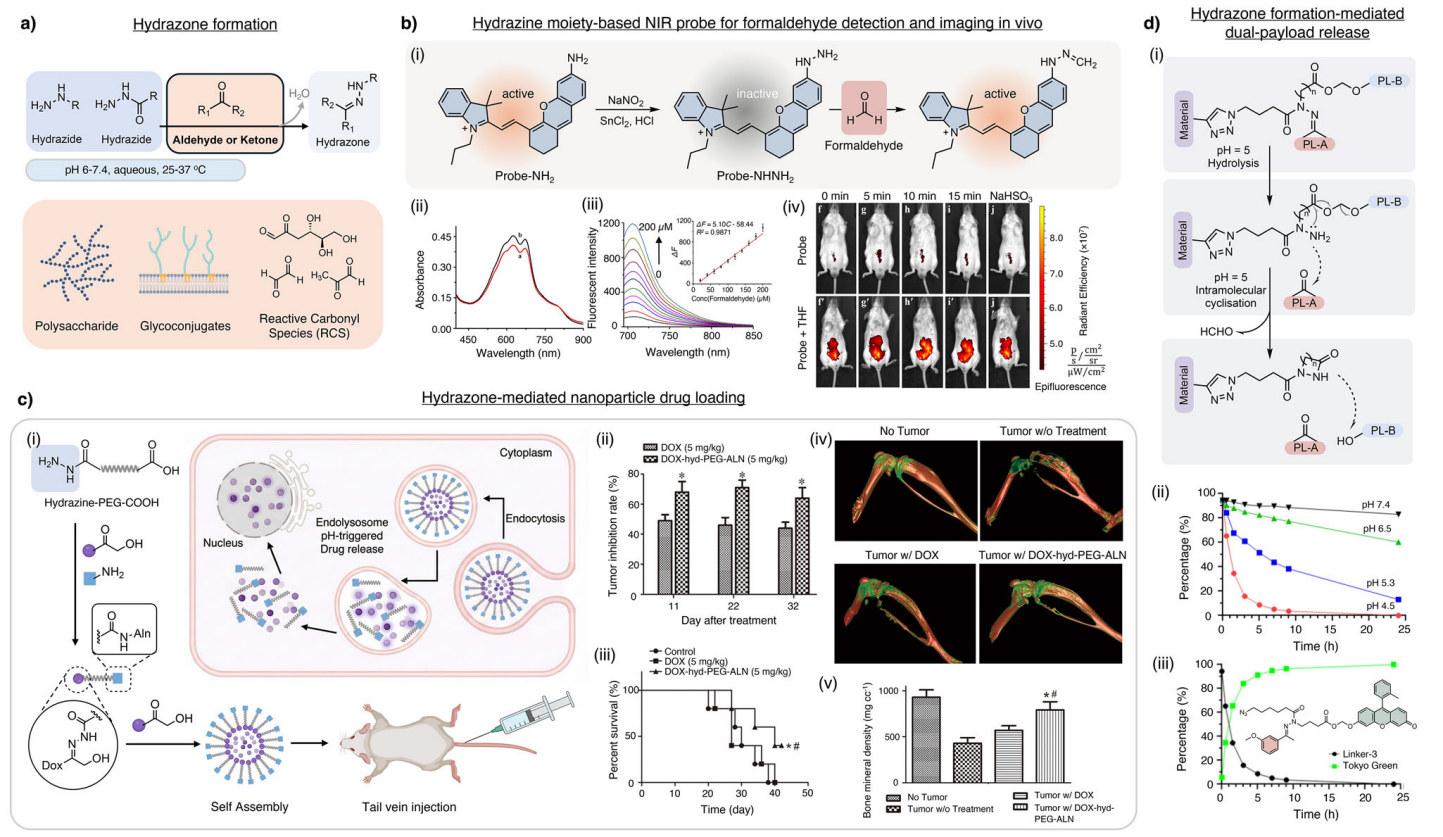
Hydrazone formation and representative biomedical applications. (a). Overall formula of hydrazone formation by carbonyl groups and hydrazine‐family nucleophiles, with typical in vivo existing carbonyl‐bearing molecules. (b). Hydrazine‐based NIR fluorescent probe for detection and in vivo imaging of endogenous formaldehyde (i). The probe showed higher absorbance in the presence of formaldehyde than in its absence (ii), and fluorescence intensity (λ_ex/em_ = 670/706 nm), correlated linearly with formaldehyde concentration (iii). In vivo, the probe enabled imaging of endogenous formaldehyde. THF: tetrahydrofolate, used to stimulate endogenous formaldehyde production. Adapted with permission. [147] Copyright 2022, American Chemical Society. (c). Hydrazone‐containing DOX‐hyd‐PEG‐ALN micelle for pH triggered intracellular drug release (i). In a bone cancer model, this system supported an enhanced tumor inhibition rate (ii), elevated survival (iii), higher bone integrity (iv, v). DOX: doxorubicin. hyd: hydrazone. PEG: polyethylene glycol. ALN: alendronate. Adapted with permission. [150] Copyright 2015, The Author(s), distributed under CC BY License. (d) Hydrazone‐mediated dual‐payload release (i). Acid‐triggered release of payload A (PL‐A) expose a hydrazine group (ii), which then promotes stoichiometric release of payload B (PL‐B) release (iii), as PL‐B exemplified by Tokyo Green. Adapted with permission. [151] Copyright 2025, The Authors, distributed under CC BY License.

Hydrazone formation is a carbonyl‐condensation reaction in which an aldehyde or ketone reacts with a hydrazine‐family nucleophile under slightly acidic to near‐physiological conditions (pH 6–7.4) to form a hydrazone (R‐C = N‐NH‐R″) linkage with concomitant loss of water (Figure 3a, Table 1) [131, 132]. Mechanistically, the carbonyl compound first undergoes nucleophilic addition by the hydrazine‐derived reagent to form a carbinolamine intermediate, then dehydrates to generate the C = N‐N linkage. In biological settings, this reaction is most relevant to aldehyde‐ or ketone‐bearing molecules, including oxidized carbohydrates [133], reactive carbonyl species (RCS) [134, 135], and engineered carbonyl handles introduced onto biomolecules for conjugation (Table 2) [136]. Compared with imine Schiff bases, hydrazones generally exhibit greater kinetic stability under aqueous conditions. Under near‐neutral conditions, uncatalyzed hydrazone formation is often considered slow, whereas favorable aromatic, neighboring carboxyl‐ or boron‐assisted systems can reach reported *k*_2_ on the order of 2–20 m ^−1^ s^−1^ (Table 3) [42, 137, 138, 139, 140, 141].

For imaging probes, hydrazone formation is widely applied to generate responsive systems in which the hydrazine‐carbonyl reaction or hydrazone‐based recognition motif directly produces a measurable optical signal [142, 143, 144]. For in vitro analysis, Gu et al. [145] demonstrated this in a lysosome‐targeting fluorescent probe for peroxynitrite imaging. The probe contained a morpholine unit for lysosomal targeting and a hydrazone response site for signal generation. This probe achieved an 83‐fold fluorescence increase, responded within 3 s, and presented a limit of detection (LOD) of 6 nm. Hydrazone formation also supported the Clustered Regularly Interspaced Short Palindromic Repeats (CRISPR) – CRISPR Associated Protein 12a (Cas12a) platform (CRISPR‐Cas12a system) for bacterial analysis. Sheng et al. [146], applied this reaction to accelerate the assembly of Cas12a activating CRISPR ribonucleic acid (crRNA) strand while improving single‐base mismatch discrimination. The resulting assay detected *Pseudomonas aeruginosa* over a linear range of 3.8 × 10^2^ to 3.8 × 10^6^ Colony‐Forming Units (CFU) mL^−1^ with an LOD of 24 CFU mL^−1^. At the in vivo level, Ding et al. [147] designed a hydrazine‐based near‐infrared (NIR) fluorescent probe for endogenous formaldehyde imaging (Figure 3b). The probe showed higher absorbance in the presence of formaldehyde than in its absence, confirming formaldehyde‐dependent optical activation. In the fluorescence spectra (λ_*ex*/*em*_ = 670/706 nm), signal intensity correlated linearly with formaldehyde concentration. Compared with the previously reported amine‐based analogue, the hydrazine‐derived probe responded about 3‐fold faster and exhibited an approximately 2.5‐fold increase of sensitivity [148]. reaching maximal signal within 10 min and an LOD of 0.68 µM. In zebrafish and mice models, the probe successfully enabled imaging of endogenous formaldehyde, including under tetrahydrofolate (THF) stimulation to increase formaldehyde production. These examples show that hydrazone formation chemistry can support imaging and biosensing across cellular, assay, and in vivo settings by coupling analyte recognition to optical or nucleic‐acid‐based signal output.

In drug delivery and therapeutic applications, hydrazone formation is used both to trap RCS and to create acid‐liable linkages for controlled payload release. As a therapeutic strategy, hydrazone formation has been used to scavenge RCS. RCS such as 4‐hydroxy‐trans‐2‐nonenal, methylglyoxal, glyoxal, and malondialdehyde are generated during oxidative stress, which can modify proteins and lipids and thereby contribute to vascular injury and atherogenic progression. Galvani et al. [131] showed that hydrazine‐containing quenchers react with biologically relevant RCS and identified hydralazine as an efficient scavenger capable of quenching all 4 species. Such RCS trapping has also been linked to antiatherogenic effects, indicating that hydrazone formation can contribute directly to disease‐modifying therapeutic action. In parallel, hydrazone formation has also been investigated to create linkages that remain relatively stable at neutral pH yet cleave in mildly acidic compartments. For example, Fu et al. [149] used hydrazone‐linked nanoscale covalent organic frameworks for doxorubicin (DOX) delivery. Their system showed nearly 6‐fold greater DOX release at pH 5.2 than at pH 7.4. It also led to intracellular nuclear accumulation after 4 h. This thereby supports selective release in acidic cancer‐cell compartments. Similar strategy was validated by Ye et al., who prepared DOX‐hydrazone‐PEG‐alendronate (DOX‐hyd‐PEG‐ALN) nanomicelles (Figure 3c). The micelles had a size of 114 nm that released DOX faster at pH 5.0 than at pH 7.4 [150]. In vivo, they produced stronger antitumor activity than free DOX in a nude‐mouse bone‐metastasis model. The tumor inhabitation rate remained consistently higher for the micelle group than for free DOX and reached about 65–70% at the late treatment stage, compared with about 45% of free DOX. The survival curve was also improved, in which mean survival increased from 30.03 ± 1.68 days of free DOX to 41.00 ± 1.31 days of micelle group. The µCT further showed that the micelles reduced bone destruction and helped preserve leg‐bone architecture. Bone mineral density remained about 780 mg cc^−1^ in the micelle group, comparted with about 580 mg cc^−1^ for free DOX and about 420 mg cc^−1^ in untreated tumor‐bearing mice. More recently, Abd‐Ellah et al. [151] further extended hydrazone chemistry to trifunctional arylhydrazone linkers for dual‐payload release (Figure 3d). In their design, acid‐triggered hydrazone cleavage released payload A (PL‐A) and simultaneously unmasked a hydrazine group. This hydrazine then attacked a second intramolecular carbonyl site and released payload B (PL‐B). For the optimized linker 3, the process was rapid at pH 4.5, with a half‐life of about 1 h. Tokyo Green was used as PL‐B to validate the release mechanism. Its appearance closely tracked linker hydrolysis, supporting sequential dual release in 1:1 stoichiometry.

In hydrogel and biomaterial formation, hydrazone formation is used to generate dynamic covalent crosslinks between hydrazide‐functionalized and carbonyl‐functionalized components [144]. This enables networks with tunable viscoelasticity under mild conditions. Richardson et al. [152] demonstrated this in cartilage tissue engineering using PEG hydrogels that contained different ratios of alkyl‐hydrazone and benzyl‐hydrazone crosslinks. By varying this ratio, they tuned the average stress‐relaxation time from 4.01 × 10^3^ to 2.78 × 10^6^ s. Chondrocytes encapsulated in the adaptable formulation with an average relaxation time of 3 days deposited substantially more matrix than those in the 100% benzyl‐hydrazone control. Collagen deposition increased by 190 ± 30%. Sulfated glycosaminoglycan deposition increased by 140% ± 20%. These results show that hydrazone‐based crosslinking can be used to tune matrix mechanics and thereby influence regenerative outcomes.

The broader biomedical use of hydrazone formation is limited by context‐dependent hydrolytic stability and incomplete bioorthogonality of hydrazine‐family reagents. Although hydrazone linkages are generally more stable than simple imines, linker cleavage can still occur before the intended intracellular or acidic trigger. In systemic settings, this may cause off‐target payload release [153, 154, 155]. Another limitation comes from competing reactions in vivo. Hydrazine derivatives can react with endogenous α‐keto acids such as pyruvate. They can also react with PLP, thereby reducing selectivity and potentially interfering with carbonyl‐dependent biological processes [156, 157]. While such carbonyl trapping can be therapeutically useful in reactive carbonyl scavenging, it is a liability in applications that require high conjugation specificity and systemic stability.

### 2.3 Boronate Esterification (Diol‐Reactive)

**FIGURE 4:**
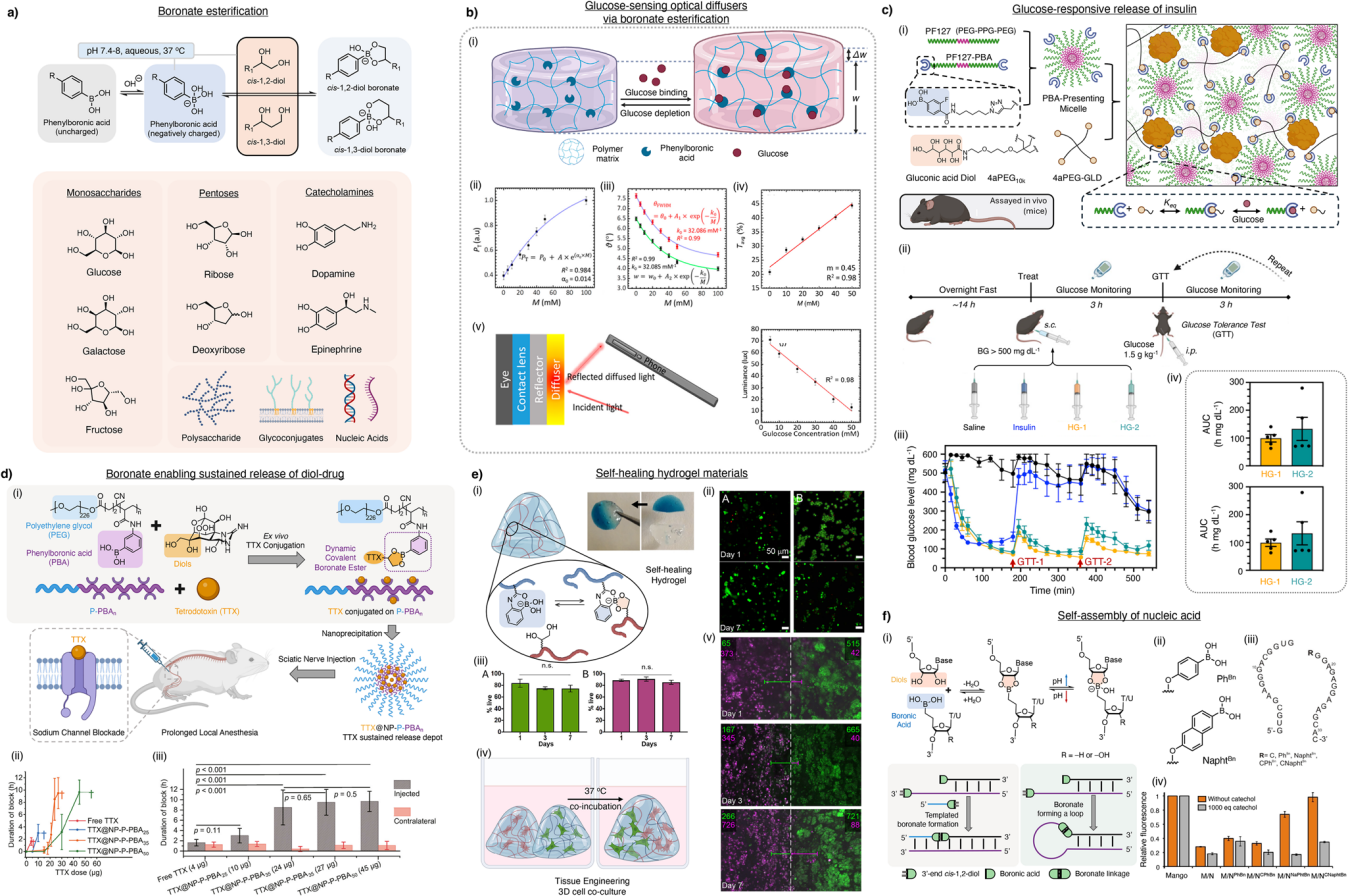
Overview of boronate esterification and representative biomedical applications. (a). overview of boronate esterification and typical endogenous diol‐bearing molecules. (b). Boronic‐acid‐functionalized hydrogel as glucose‐sensing optical diffusers (i). Glucose binding alters Donnan osmotic pressure (*P*_T_, ii), contact angle (ϑ, iii), and light transmission (*T*_avg_, %, iv) correlated to glucose concentration. The sensor was further incorporated to a phone‐based readout device (v). Adapted with permission. [190] Copyright 2018, American Chemical Society, distributed under CC BY License. (c). PBA‐diol‐crosslinked hydrogel functions for glucose‐responsive release of encapsulated insulin. In a GTT mouse model (ii), the hydrogels HG‐1 and HG‐2 provided glucose control in two consecutive GTT challenges (iii), as reflected by reduce blood‐glucose AUC at 100 h mg dL^−1^ through 2 GTTs (iv). GTT, glucose tolerance test. AUC, area under curve. Adapted with permission. [196] Copyright 2022, American Chemical Society. (d). PBA‐functionalized drug depot for diol‐bearing drug (e.g., TTX) delivery in sustained mode (i). This formulation can provide nerve blocks up to 10 h (ii‐iii). Adapted with permission. [198] Copyright 2024, Wiley‐VCH GmbH. (e). Self‐healing hydrogels crosslinked with reversible boronate ester formation between boronic acids and diols (i). The hydrogels showed excellent cell viability (ii‐iii) and supported 3D cell co‐culture through dynamic merging of two cell‐laden hydrogel compartments (iv‐v). Adapted with permission. [199] Copyright 2018, American Chemical Society. (f) Boronic‐acid–mediated RNA self‐assembly through cis‐diol esterification, restoring RNA structure and functionality. In this context, Ph^Bn^‐ and Napht^Bn^‐modified RNA fragments (ii, iii) were used for Mango RNA reconstitution and detection (iv). Adapted with permission. [200] Copyright 2023, The Authors. Chemistry – A European Journal published by Wiley‐VCH GmbH, distributed under CC BY License.

Boronate esterification is a reversible, catalyst‐free condensation in which a boronic acid, most commonly an arylboronic acid such as phenylboronic acid (PBA), reacts with a *cis*‐1,2‐diol or *cis*‐1,3‐diol to form a cyclic tetrahedral boronate ester under physiological conditions (Figure 4a, Table 1) [158, 159, 160]. The equilibrium of this dynamic covalent bond depends strongly on pH, the pK_a_ of the boronic acid, and the geometry and electronic properties of the diol partner. In biological settings, relevant diol‐bearing targets include carbohydrates such as glucose and fructose [161, 162, 163] glycan motifs on glycoconjugates [164, 165, 166], catecholamines [167, 168, 169], and ribose‐containing biomolecules [170, 171] (Table 2). Practical complexation near physiological pH is often improved through boronic‐acid design, including electron‐withdrawing substituents (e.g., 3‐fluoro‐PBA, FPBA) [172, 173], ortho‐amine motifs (e.g., 2‐amino‐PBA) [174, 175], benzoxaboroles [176, 177], and diboronic‐acid architectures [163, 178], which can lower the effective pK_a_ and enhance binding affinity or selectivity [179, 180, 181, 182]. Kinetically, boronate esterification is generally fast and reversible, with reported *k*_2_ of 1–50 m ^−1^ s^−1^ for plain PBA [183, 184], and 10^2^–10^3^ m ^−1^ s^−1^ for pK_a_‐engineered boronic acids (Table 3) [185, 186, 187, 188].

In sensing and diagnostic probes, boronate esterification is most prominently used for non‐enzymatic glucose sensing, while also enabling selective detection for other *cis*‐diol‐ or catechol‐bearing biomolecules [161, 162, 163, 189]. For example, Elsherif et al. [190] developed a PBA‐functionalized hydrogel optical diffuser for glucose sensing (Figure 4b). In this design, glucose reacted with immobilized PBA groups. This generated boronated anions and increased Donnan osmotic pressure, and thereby caused hydrogel swelling that altered light transmission. The resulting sensor showed a linear response over 0–50 mm with a sensitivity of 11.6 µW mm ^−1^, and reached saturation in less than 60 min. The system was further integrated into a contact‐lens format and read out using a smartphone photodiode, supporting wearable glucose monitoring under physiological conditions. Beyond glucose, boronate esterification has also been applied for catecholamine recognition. Godoy‐Reyes et al. [191] developed a gold‐nanoparticle‐based colorimetric probe for norepinephrine detection. The system achieved a linear response from 0 to 1 µM and LODs of 0.07 µM in water and 0.09 µM in synthetic urine. Likewise, Devi et al. [192] reported boronic‐acid‐functionalized tungsten disulfide quantum dots as a fluorescence probe for dopamine. The probe exhibited a linear range of 0.05‐100 µM and an LOD of 0.01 µM. Together, these studies show that boronate esterification can support diagnostic probe design not only for glucose sensing, but also for selective recognition of catechol‐bearing biomolecules through rapid and reversible covalent binding.

In drug delivery and therapeutic applications, boronate esterification is widely used to create reversible diol‐responsive linkages and crosslinks for glucose‐triggered release and depot‐type delivery. Early studies established that boronate esterification with glucose can form injectable hydrogels with glucose‐responsive behaviors including glucose‐triggered swelling, sol–gel transitions, or cargo release [159, 160, 193, 194, 195]. These studies established the feasibility of boronate esterification for glucose‐regulated therapeutic systems. Xian et al. [196] developed glucose‐responsive injectable thermogels by dynamically crosslinking PBA‐functionalized Pluronic F127 micelles with 4‐arm‐PEG bearing gluconic‐acid diol termini (4aPEG‐GLD, Figure 4c). In this system, 4aPEG‐GLD initially occupies the PBA groups to form boronate ester crosslinks. Glucose then competitively displaced these diol interactions, weakened the network, and thereby promoted insulin payload release. In diabetic mice, a single subcutaneous injection of insulin‐loaded thermogel maintained glucose control through two intraperitoneal glucose tolerance tests (GTTs, 100 µL glucose, 1.25 g kg^−1^ each). After each challenge, blood glucose rose to about 200 mg dL^−1^ and then returned to a normoglycemic range. The material retained responsiveness through the second GTT, which occurred about 9 h after treatment. Xiang et al. [197] subsequently refined this strategy incorporating diboronate (DiPBA) crosslinking. This design strengthened glucose responsiveness and glucose specificity. In diabetic mice subjected to the same two GTTs, DiPBA formulation decreased blood‐glucose AUC by 2‐fold after the second GTT compared to the FPBA formulation. More recently, Xian et al. [178] further simplified the system by conjugating a *cis*‐diol to insulin and loading it into hyaluronic acid (HA)‐DiPBA carrier, thereby replacing the traditional 4aPEG‐GLD stabilizer. The resulting system is able to achieve a 1.7‐fold increase of serum insulin after their first GTT and restore the blood glucose to pre‐dosing baseline of 66 mg dL^−1^ by 3 h. Beyond glucose‐responsive insulin delivery, Xue et al. [198] used boronate esterification to conjugate tetrodotoxin (TTX) through its 1,2‐diols to PBA‐containing polymers, generating polymeric NPs with >90% encapsulation efficiency (Figure 4d). In vivo, perineural injection prolonged nerve block to 9.7 ± 2.0 h from 1.6 ± 0.6 h by free TTX. Co‐delivery of dexamethasone, which also bears diol groups, further extended nerve block to 21.8 ± 5.1 h while reducing systemic toxicity.

In hydrogel and biomaterial formation, boronate esterification is used to generate dynamic crosslinks under mild aqueous conditions [179, 180, 181]. This chemistry supports both self‐healing networks and bioactive scaffolds. Smithmyer et al. [199] demonstrated this in boronic‐acid‐based hydrogels for 3D co‐cultures. In their design, polymers bearing 2‐acrylamidophenylboronic acid (2APBA) were crosslinked with poly(vinyl alcohol) through reversible boronate ester formation (Figure 4e). The hydrogels formed and healed in PBS, serum‐free medium, and serum‐containing cell culture medium. They also supported encapsulation of MDA‐MB‐231 breast cancer cells and CCL151 fibroblasts with nice viability over 7 days. The hydrogel exhibited self‐healing behavior, which enabled fusion of cell‐laden blocks into layered 3D co‐cultures. Thibault et al. [195] prepared freeze‐dried composite scaffolds from PBA‐functionalized chitosan and Bioglass 45S5. These scaffolds exhibited 3D interconnected porosity and no toxicity toward mouse Sertoli cells (TM4), human embryo kidney 293 cells, or human bone marrow stromal cells (HS‐5), supporting the use of boronate esterification for bone tissue engineering. Collectively, these studies show that boronate esterification can support regenerative medicine both by enabling dynamic self‐healing networks and by improving the bioactivity of tissue‐engineering scaffolds.

In synthetic biology and molecular reconstitution, boronate esterification has been explored as a catalyst‐free strategy for reversible RNA assembly. Lelièvre–Büttner et al. [200] showed that a 5’‐boronic‐acid‐modified RNA fragment with the 3’‐terminal ribose *cis*‐diol for another fragment to form an internucleosidic boronate ester (Figure 4f). This reaction promoted assembly of split functional RNAs. Using this strategy, the investigators restored activity in selected split variants of the hairpin ribozyme and recovered function in split Mango aptamers. This discovery showed that boronate esterification can serve to form reversible surrogates of phosphodiester linkages in functional RNA architectures, supporting programmable nucleic acid assembly for synthetic biology.

The broader biomedical use of boronate esterification is mainly limited by equilibrium sensitivity, oxidative liability of many arylboronic acids, and the mechanical weakness of networks crosslinked solely through this reaction. In biomaterials and depot systems, boronate‐diol crosslinks alone may not provide sufficient structural persistence for prolonged function. A related tradeoff was observed in the simplified HA‐DiPBA system reported by Xian et al., where eliminating the 4aPEG‐GLD crosslinker shortened functional persistence and compromised responsiveness during the second GTT, despite preserving glucose‐triggered insulin release during the first GTT [198]. In addition, many arylboronic acids are susceptible to oxidation by ROS, which can compromise long‐term biological performance and potentially introduce cytotoxic phenols into the metabolism [201]. Although newer boronic‐acid chemotypes have been developed to improve oxidative stability, such structural modification also compromised pK_a_ and diol‐binding affinity; for example, the oxidatively stabilized variant showed a D‐glucose binding constant of 1.7 ± 0.1 m ^−1^, compared with 5 ± 1 m ^−1^ of PBA and 28 ± 4 m ^−1^ of benzoxaborole. Thus, oxidative liability remains a substantial challenge for long‐term biomedical applications [202].

### 2.4 Thiol‐Michael Addition (Thiol‐Reactive)

**FIGURE 5:**
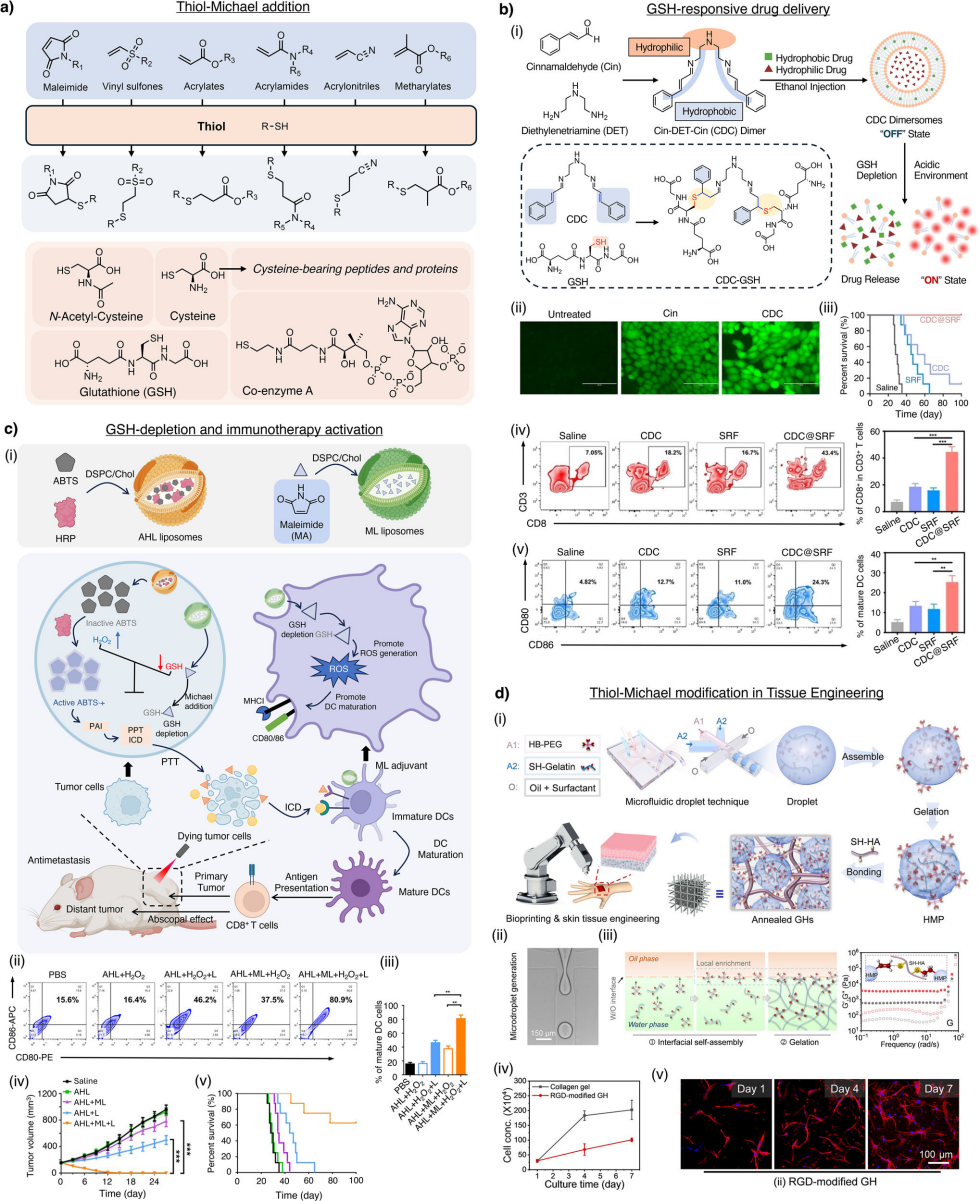
Thiol‐Michael addition reactions and their biomedical applications. (a). Overall formula of Thiol‐Michael addition reactions, and representative thiol‐bearing biomolecules existing in vivo. (b). Cinnamaldehyde‐incorporated CDC dimersomes and its glutathione (GSH)‐responsive sorafenib (SRF) release upon thiol‐Michael addition (i). CDC deplete intracellular GSH, disrupt redox balance, and promote ROS accumulation for killing of tumor cells (ii). Together with SRF, the formulation significantly enhanced in survival in cancer animal model (iii) with enhanced CD8^+^ T cell infiltration (iv) and mature dendritic cell (DC) maturation (v). Adapted with permission. [211] Copyright 2022, Wiley‐VCH GmbH. (c). Maleimide‐loaded liposomes co‐administered with horseradish peroxidase (HRP) and 2,2′‐Azino‐bis(3‐ethylbenzothiazoline‐6‐sulfonic acid) (ABTS) for GSH depletion and immunotherapy activation. This strategy enhanced the mature DC cell amount (ii, iii), promoted tumor inhibition (iv), and improved animal survival (v). PTT, photothermal therapy. ICD, immunogenic cell death. Adapted with permission. [212] Copyright 2020, The American Association for the Advancement of Science, licensed under the Creative Commons CC BY‐NC License. Non‐commercial use only. (d) Thiol‐Michael modified reinforced granular hydrogel scaffolds for skin tissue engineering (i). The granules are prepared in vitro (ii) while undergo in vivo self‐assembly and gelation after administration (iii), which supports proliferation of human dermal fibroblast cells (iv, v). Adapted with permission. [213] Copyright 2025, The Author(s). Advanced Science published by Wiley‐VCH GmbH, distributed under CC BY License.

Thiol‐Michael addition is a 1,4‐conjugate addition in which a thiol nucleophile reacts with an electron‐deficient α,β‐unsaturated acceptor (e.g., enones, acrylates) under mild aqueous conditions (Figure 5a) [203]. Mechanistically, thiol‐Michael addition generally proceeds through nucleophilic attack of a thiolate at the β‐carbon of the activated alkene, followed by protonation to yield the thioether adduct. In biomolecular contexts, the principal acceptors include maleimides, acrylates, acrylamides, vinyl sulfones, and fumarates. Relevant thiol‐bearing partners include cysteine residues in proteins and peptides as well as small‐molecule thiols such as glutathione (GSH), so both desired conjugation and competing endogenous capture can occur under physiological conditions (Figure 5a, Table 2) [203, 204]. Thiol‐Michael addition is kinetically rapid, with reported *k*_2_ on the order 10–10^4^ m ^−1^ s^−1^ (Table 3) [48, 203, 205, 206]. The reaction shows strong thiol preference around neutral pH. However, its selectivity and stability are governed by the tradeoff between electrophile reactivity and susceptibility to exchange, hydrolysis, or competing nucleophiles [207].

In targeted cancer therapy, thiol‐Michael addition is most prominently used in targeted covalent inhibitors. In these systems, a moderately electrophilic α,β‐unsaturated acceptor reacts with a strategically positioned cysteine residue to achieve durable and selective target engagement. Clinically approved exemplars such as ibrutinib, osimertinib, sotorasib, and adagrasib illustrate that acrylamide‐type warheads can be tuned to react only after productive noncovalent binding, rather than through indiscriminate bulk thiol reactivity [204]. A particularly important case is an oncogenic Kirsten rat sarcoma (KRAS) mutant, KRAS^G12C^, in which glycine‐12 is replaced by cysteine, creating a targetable thiol for covalent inhibitor design. Huynh et al. [208] showed that the KRAS^G12C^ thiol has a depressed pK_a_ of 7.6 ± 0.4. This increases the fraction of reactive thiolate at physiological pH and make the site more susceptible to thiol‐Michael addition. Using ARS‐853, an acrylamide‐bearing covalent inhibitor, they further found that covalent inhibition was strongly pH dependent. The *k_inact_*/*K_i_* values, which reflect overall covalent inhibition efficiency, reached 510 ± 75 m ^−1^ s^−1^ at 20°C and pH 7.5. They also showed that reversible oxidation or glutathionylation of the mutant cysteine interfered with covalent binding. These findings show that thiol‐Michael chemistry in covalent oncology drugs depends not only on warhead electrophilicity, but also on the ionization state and accessibility of the target cysteine.

In drug delivery and therapeutic applications, thiol‐Michael addition is used both to enable GSH‐responsive prodrug activation and for delivery systems that exploit intracellular thiol chemistry for controlled release or redox modulation. Early work established this concept through GSH‐dependent thiopurine prodrugs [209, 210]. One example is *cis*‐3‐(9*H*‐purin‐6‐ylthio)acylic acid (PTA), which acts as an α,β‐unsaturated acceptor. PTA is converted to 6‐mercaptopurine (6‐MP) through GSH‐dependent pathways in vitro and in vivo. After *i.p*. administration of 100 mg kg^−1^ PTA in rats, 6‐MP was detected in urine without apparent hepatic or kidney toxicity [209]. More recently, Zhou et al. [211] used cinnamaldehyde dimers (CDC) that undergo thiol‐Michael addition with intracellular GSH, thereby depleting GSH while triggering dimersome breakdown and sorafenib (SRF) release (Figure 5b). GSH is a major intracellular antioxidant in tumor cells that helps maintain redox balance. CDC directly depleted existing intracellular GSH, whereas SRF further prevented GSH resynthesis by inhibiting the system $x_{c}^{-}$ pathway. Together, these effects impaired antioxidant defense, promoted lipid ROS accumulation, and thereby enhanced ferroptosis. In vivo, this formulation promoted maturation of dendritic cells (DCs) to 24.3% and increased tumor‐infiltrating CD8^+^ T cells to 43.4%, and all treated mice survived to 100 days, whereas most mice in the CDC‐ or SRF‐only groups died within 100 days. These studies demonstrate thiol‐Michael addition underpin GSH responsive drug delivery covering from prodrugs to depot carriers.

In immunotherapy, thiol‐Michael addition has also been used to deplete intracellular GSH as an adjuvant strategy to amplify antitumor immunity. For example, Zhou et al. [212] reported maleimide‐functionalized liposomes that react with GSH through thiol‐Michael addition (Figure 5c). This adjuvant system lowered tumoral GSH and broke the redox balance in tumor cells. This relieved redox suppression promoted activation of 2,2′‐azino‐bis(3‐ethylbenzothiazoline‐6‐sulfonic acid) (ABTS) to radical form ABTS•^+^ for photoacoustic imaging‐guided photothermal therapy (PTT), which enhanced immunogenic cell death. Concurrently, GSH depletion in DCs increased ROS and promoted DC maturation, thereby amplifying antitumor immune activation. In vivo, these liposomes reduced intratumoral GSH to 43% of the saline‐treated group, increased dendritic‐cell maturation to 80.9% after laser treatment, and raised intratumoral CD8^+^ T‐cell infiltration to 53%. Functionally, the treatment also suppressed distant untreated tumors and reduced lung metastatic nodules from about 150 per mouse in the saline group to fewer than 5 after therapy. These results show that thiol‐Michael chemistry can participate in activation of immunotherapy via modulating GSH levels in diseased sites.

In hydrogel and biomaterial formation, thiol‐Michael addition is used to generate cytocompatible C‐S crosslinks under mild conditions. This chemistry enables injectable materials, tissue adhesives, and scaffolds with tunable mechanics. For example, Zhang et al. [213] demonstrated this in reinforced granular hydrogel scaffolds for skin tissue engineering (Figure 5d). In their design, thiol‐Michael chemistry was used to integrate the network and tune scaffold composition. The optimized scaffold showed a compressive modulus of 41.4 ± 3.7 kPa, compared with 11.4 ± 1.2 kPa for the non‐reinforced control. It also maintained high porosity and injectability. The scaffold was biocompatible and showed minimal cytotoxicity. Human dermal fibroblasts proliferated within the scaffold and formed network structures. Although proliferation was faster in collagen hydrogel, the granular scaffold retained its original dimensions and showed much stronger resistance to contraction during long‐term culture. The system also enabled construction of full‐thickness engineered skin tissue while minimizing contraction during long‐term culture. Lamas et al. [214] used base‐triggered thiol/vinyl ether Michael addition to prepare thermoswitchable adhesives with well‐defined microphase‐separated structures. This result shows that thiol‐Michael addition can also be used to build reversible adhesive biomaterials with tunable thermal and mechanical behavior. Together, these studies show that thiol‐Michael addition can support biomaterials design not only through efficient network formation, but also through control over scaffold mechanics and tissue‐facing function.

The broader biomedical use of thiol‐Michael addition is limited mainly by the need to balance electrophile reactivity against selectivity, and by the fact that some thiol‐Michael adducts do not remain fully stable in complex biological environments. Reactive Michael acceptors may be consumed by endogenous thiols such as GSH or serum proteins before reaching the intended target [204, 215]. In hydrogels and biomaterials, thiol‐maleimide and related linkages can further undergo exchange, hydrolysis, retro‐Michael cleavage, or radical‐mediated degradation. These susceptible progresses may compromise long‐term network integrity under physiological or ROS‐rich conditions [216, 217, 218, 219]. Accordingly, durable biomedical use often depends on stabilized maleimide designs, alternative acceptors, or scaffold‐level strategies that reduce unwanted exchange and degradation [207, 218, 219, 220, 221, 222, 223].

### 2.5 Bioorthogonal Chemistry

**FIGURE 6:**
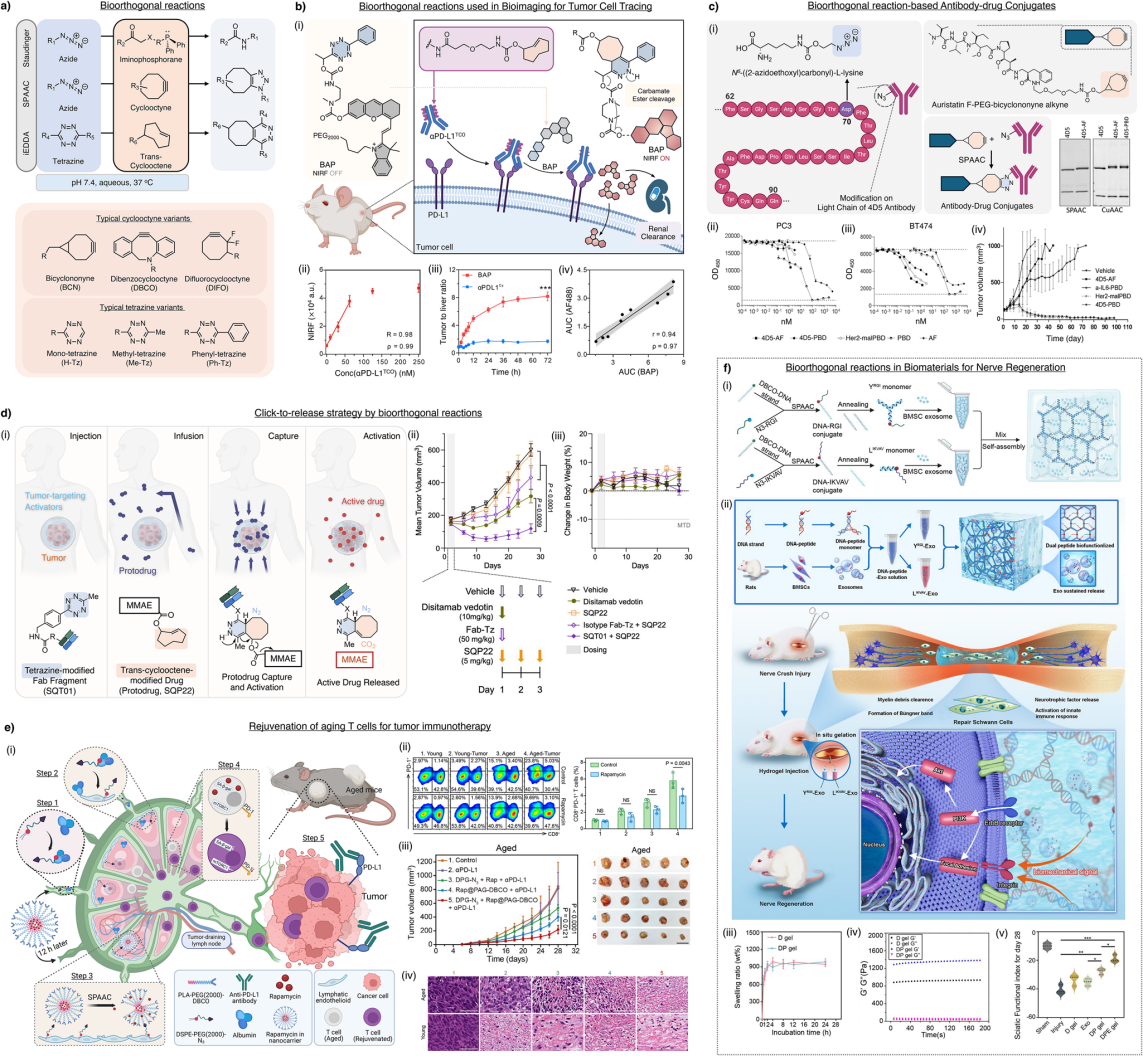
Bioorthogonal reactions and representative biomedical applications. (a). Typical types of bioorthogonal reactions, including Staudinger ligation, strain‐promoted azide‐alkyne cycloaddtion (SPAAC), inverse electron‐demanded Diels‐Alder reaction (iEDDA). (b) Tumor bioimaging through iEDDA between a trans‐cyclooctene (TCO)‐modified anti‐PD‐L1 antibody and a tetrazine‐containing bioorthogonally activatable near‐infrared fluorescent probe (BAP, i). This system produced correlated NIR signal (ii), excellent tumor‐to‐background ratio (iii). Compared with AF488‐based immunostaining, this system showed increased AUC by 1.9 to 2.3 folds (iv). Adapted with permission. [244] Copyright 2025, Wiley‐VCH GmbH. (c). Site‐specific antibody‐drug conjugates generated by SPAAC reaction between Lys‐70 azide‐modified 4D5 antibody and BCN‐modified anti‐tumor drugs auristatin F (AF) and pyrrolobenzodiazepine (PDB) dimer (i). This system preserved low functionality to Her2‐low cancer cells (PT3, ii) but significantly improved in vitro cytotoxicity toward Her2‐high cancer cell lines (BT474, iii). In a mice model bearing BT474 xenograft, the optimal system succeeded in reducing the tumor volume in vivo (iv). CuAAC, copper(I)‐catalyzed azide‐alkyne cycloaddition. SPAAC, strain‐promoted azide‐alkyne cycloaddition. Adapted with permission. [246] Copyright 2015, American Chemical Society. (d). “Click‐to‐release” monomethyl auristatin E (MMAE) protodrug for controlled intratumoral drug release upon bioorthogonal activation (i). This drug delivery approach significantly inhibited tumor progression (ii) with minimal body weight alteration (iii).Adapted with permission. [248] Copyright 2023, American Chemical Society, distributed under the CC BY 4.0 License. Non‐commercial use only. (e). Bioorthogonal click‐chemistry‐assisted rejuvenation of aged T cells renders aged mice responsive to tumor immunotherapy (i). The routine immunotherapy offers significantly enhanced CD8^+^ PD‐1^+^ T cell level (ii), slowed‐down tumor progression as in tumor volume (iii) and H&E staining assays (iv). PLA, Polylactic acid. DBCO, Dibenzocyclooctyne. PD, Programmed Cell Death Protein. Adapted with permission. [249] Copyright 2025, American Chemical Society. f) Programmable DNA–peptide conjugated hydrogel for peripheral nerve regeneration via SPAAC between DBCO‐modified DNA monomers and azide‐functionalized peptides (i, ii). The hydrogel provides rapid swelling (iii), increase storage modulus (iv), and significantly elevated nerve repair functionality (v). Adapted with permission. [250] Copyright 2025, Wiley‐VCH GmbH.

Bioorthogonal reactions are high‐yielding, rapid, and mutually selective reactions that proceed in living systems without perturbing native physiology. The modern development of this field is most closely associated with the pioneering work of Carolyn R. Bertozzi, who established bioorthogonal chemistry as a practical strategy for carrying out selective ligation in living systems [224, 225, 226]. Starting from early studies on chemical reporters in glycobiology, her work showed that abiotic functional groups (e.g., azides) could be introduced into biomolecules, and then selectively coupled in complex biological environments without disrupting native cellular chemistry. Although bioorthogonal chemistry is often discussed within the broader framework of click chemistry, only a subset of click reactions satisfies the stringent criteria of true in vivo bioorthogonality [11, 48]. The bioorthogonal principal families discussed here include Staudinger ligation [227, 228], strain‐promoted azide–alkyne cycloaddition (SPAAC) [229, 230, 231, 232], and the tetrazine ligation based on the inverse electron‐demand Diels–Alder reaction (iEDDA) [233, 234, 235] (Figure 6a, Table 1). These reactions rely on complementary reactive partners that are absent from or minimally interfering with endogenous biochemistry, such as azides, cyclooctynes, tetrazines, and *trans*‐cyclooctenes (TCOs, Figure 6a, Table 2). Their kinetics span a broad range under near‐physiological conditions. Staudinger ligation is considered relatively slow, whose *k*_2_ typically lies on the order of 10^−3^ M^−1^ s^−1^ [48, 236]; SPAAC generally proceeds from 10^−2^ to 10^2^ m ^−1^ s^−1^ [48, 237, 238], and tetrazine‐TCO iEDDA is widely regarded as among the most instantaneous bioorthogonal ligations, with reported rate constants *k*_2_ = 10^3^–10^6^ m ^−^^1^ s^−^^1^ [48, 239, 240]. This large kinetic spread is important in practice, because faster ligations are favored when short residence times or low local concentrations demand rapid capture [233, 239, 241].

In imaging and diagnostic probes, bioorthogonal reactions are widely used to achieve selective signal turn‐on in living systems. They are especially useful when rapid ligation and low background are required. Early fluorogenic SPAAC probes established that azide‐cyclooctyne ligation can directly unmask fluorescence in a catalyst‐free manner [230, 242, 243]. More recently, Liu et al. [244] extended this concept to immune‐checkpoint imaging of cancer with an NIR bioorthogonally activatable fluorescence probe for programmed cell death ligand 1 (PD‐L1) (Figure 6b). In their design, the hemicyanine fluorophore was caged by tetrazine and remained weakly fluorescent. A TCO‐tagged anti‐PD‐L1 antibody (⍺PDL1‐TCO) first bound tumor PD‐L1. The tetrazine‐containing bioorhogonally activatable probe (BAP) was then administered. iEDDA reaction with the TCO groups cleaved the tetrazine cage and released the uncaged hemicyanine, which turned on NIR fluorescence (NIRF). Upon iEDDA‐mediated activation, the probe showed a 25‐fold fluorescence enhancement. Its signal increased linearly with ⍺PDL1‐TCO concentration over 0 to 6.25 nM, with an LOD of 3.48 nM. In vivo, tumor fluorescence peaked at 6 h with a signal‐to‐background ratio of 9.5. The tumor‐to‐liver ratio increased over time and reached 8.6 at 72 h, supporting high‐contrast in vivo imaging. The area‐under‐curve (AUC) values were found 2.5‐, 1.9‐, and 2.3‐fold higher than those from AF488‐based immunostaining after 0, 1, and 2 doses of cisplatin. Zhou et al. [245] further extended bioorthogonal imaging to dynamic metabolic tracking by designing a bioluminescent probe for glutamine uptake mediated by alanine, serine, cysteine transporter 2 (ASCT2) in living tumors. The system reached maximum signal within 14 min. In vivo, the tumor bioluminescence was abolished by the ASCT2 inhibitor V9302, confirming transporter‐dependent imaging. Together, these studies show that bioorthogonal ligation can support biomedical imaging through both selective labeling and real‐time signal activation in vivo.

In the context of drug delivery, bioorthogonal reactions are used both to construct site‐defined antibody conjugates and to decouple distribution from activation for localized drug release. A particularly important application is the construction of site‐defined ADCs. In these systems, bioorthogonal reactive handles enable controlled payload attachment and reduce the heterogeneity associated with conventional lysine‐ or cysteine‐based conjugation. [11, 47, 246]. For example, VanBrunt et al. [246] demonstrated this by genetically encoding N6‐((2‐azidorthoxyl)carbonyl)‐L‐lysine into an antibody at defined positions. The azide‐bearing antibody was then reacted with alkyne‐ or cyclooctyne‐bearing toxins via click cycloadditions to form a stable triazole linkages (Figure 6c). This strategy gave >95% conjugation efficiency and drug‐to‐antibody ratios (DAR) >1.9. The main antibody used was 4D5, an anti‐Her2/neu antibody. The payloads included auristatin F (AF) and a pyrrolobenzodiazepine (PBD) dimer. In vitro, the 4D5‐AF conjugates were strongly cytotoxic to Her2‐high BT474 cells (i.e., half‐maximal inhibitory concentration [IC_50_] of 0.101 ± 0.032 nM) but showed compromised activity in Her2‐low PC3 cells (unable to quantify IC_50_), supporting target‐dependent ADC activity. In vivo, the 4D5‐PBD conjugate was evaluated in BT474 xenografts in CB.17 SCID mice. Tumor volume was monitored for 90 days after weekly intravenous dosing for 3 weeks at 1 mg kg^−1^. Both 4D5‐PBD and the conventional Her2‐malPBD comparator produced sustained tumor regressions. By contrast, vehicle, 4D5‐AF, and the control antibody interleukin (IL) 6 conjugate a‐IL6‐PBD showed continued tumor growth to roughly 1000 mm^3^. Bruins et al. [247] developed a non‐genetic chemoenzymatic tyrosine‐click strategy for antibody conjugation. Their method first removed the native N297 glycan from human immunoglobulin G1 with peptide‐N‐glycosidase F, which exposed a nearby Fc‐domain tyrosine. Mushroom tyrosinase then oxidized this tyrosine to an ortho‐quinone, which reacted with a strained alkyne or alkene through strain‐promoted oxidation‐controlled quinone cycloaddition. This method produced homogenous DAR2 and DAR4 ADCs with monomethyl auristatin E (MMAE) or PBD dimer payloads. In addition, bioorthogonal reactions have also enabled spatially controlled protodrug activation. A direct example was reported by McFarland et al. [248], who developed an iEDDA click‐activated MMAE protodrug platform (Figure 6d). The protodrug, SQP22, is a TCO‐modified MMAE derivative. By itself, SQP22 was strongly attenuated, showing >50‐fold lower cytotoxicity than free MMAE. After reaction with tetrazine, active MMAE was efficiently released and the potency was restored. The targeting agent SQT01 was a HER2‐binding Fab fragment conjugated with tetrazine. Its role was to localize tetrazine at HER2‐postive tumors, so that systemically administered SQP22 could be activated selectively at the tumor site. In the Karpas 299 xenograft model, protodrug SQP22 activated via intratumoral tetrazine pre‐installed by SQT01 suppressed HER2‐postive NCI‐N87 xenografts more effectively than SQP22 alone, disitamab vedotin (HER2‐targeting MMAE ADC), or the isotype Fab‐Tz control.

For immunotherapy, bioorthogonal reactions have been used to enhance immune‐checkpoint blockade by directing immunomodulators to lymphoid sites control antitumor T‐cell priming. Bai et al. [249] developed a tumor‐draining lymph node (TdLN)‐targeted delivery system based on azide‐dibenzocyclooctyne (DBCO) SPAAC chemistry. In this strategy, azide‐modified DSPE‐PEG_2000_ (DPG‐N3) was first injected intradermally, then it exploited interstitial albumin to accumulate in TdLNs. The albumin‐DPG‐N3 is thereby anchored into lymphatic endothelial‐cell membranes. After 12 h, DBCO‐modified rapamycin‐loaded polylactic acid (PLA)‐PEG micelles (Rap@PAG‐DBCO) were injected at the same site. The bioorthogonal click reaction then enriched rapamycin specifically in TdLNs, where it rejuvenated aged CD8^+^ T cells and improved the response to αPD‐L1 therapy. Functionally, the expression of PD‐1 on intratumoral CD8^+^ T cells was reduced by 1.4‐fold and 2.1‐fold relative to the two partial‐control formulations. In the aged MC38 tumor model, this treatment produced the strongest tumor‐volume control in aged mice, whereas αPD‐L1 alone was insufficient and the same delivery strategy added little benefit in young mice. Histologically, only tumors from aged mice treated with the TdLN delivery system plus αPD‐L1 showed extensive necrosis by hematoxxylin and eosin (H&E) staining. At the safety level, the regimen caused no noticeable body‐weight loss and no evident pathological damage in major organs, indicating good tolerability. Together, these results show that SPAAC‐assisted TdLN targeting can rejuvenate aged tumor‐reactive CD8^+^ T cells and restore sensitivity to immune‐checkpoint blockade in aged mice.

In hydrogel and biomaterial formation, bioorthogonal reactions are used to assemble multifunctional networks under mild conditions while preserving the activity of embedded biological signals. Wei et al. [250] demonstrated this with a programmable DNA‐peptide conjugated hydrogel for peripheral nerve regeneration. In their design, SPAAC was used to couple DBCO‐modified DNA monomers with azide‐functionalized peptides. The resulting hydrogel gelled within 10 s and showed a higher storage modulus (1347.9 Pa) than the unmodified DNA hydrogel (916.3 Pa). Biologically, the system promoted Schwann‐cell reprogramming, proliferation, and migration through the Nrg1/ErbB/PI3K/Akt pathway. It also enhanced neuronal axon outgrowth and endothelial tube formation. In a rat sciatic nerve crush model, the hydrogel gelled in situ and promoted nerve regeneration and functional recovery. These results show that SPAAC can enable multifunctional regenerative hydrogels with both mechanical and biological programmability. Likewise, Shi et al. [251] used SPAAC to construct extravesicular vesicle (EV)‐crosslinked hydrogels. The hydrogels showed that this design dramatically accelerated stress relaxation. The half‐relaxation time decreased from 1429 s in conventional control to 4.122 s in the EV‐crosslinked hydrogel. Together, these studies show that bioorthogonal ligation can support biofabrication through rapid and cytocompatible hydrogel assembly while enabling precise incorporation of bioactive components into regenerative materials.

The broader biomedical translation of bioorthogonal chemistry is limited mainly by mismatch between the delivery and pharmacokinetics, the tradeoff between handle reactivity and stability, and chemistry‐specific biological liabilities. In vivo, the two reaction partners must reach the same site at sufficient concentration and within overlapping time windows. This is especially challenging in pretargeting systems and often necessitates clearing or masking strategies [253, 254]. At the same time, highly reactive motifs can suffer decreased stability in biological media, which creates a practical tradeoff between fast ligation and sufficient circulation lifetime [255, 256]. Some bioorthogonal systems also show scaffold‐dependent biocompatibility issues. For example, certain strained‐alkyne formulations have shown complement‐related compromise in biocompatibility [257]. In addition, TCO can undergo *cis–trans* isomerization in vivo, reducing effective iEDDA capture [258]. These constraints mean that successful in vivo application often depends not only on reaction kinetics, but also on careful reagent engineering and system‐level optimization.

## 3 Spontaneous Non‐Covalent Physical Interactions

In addition to covalent reactions, non‐covalent, physical binding interactions that proceed autonomously under physiological conditions are central to structural stabilization and molecular recognition in vivo [259, 260]. We therefore summarize four representative modes: hydrophobic interactions, hydrogen bonding/*π*–*π* stacking, ionic interactions, and host–guest recognition, which serves as complementary design tools for diagnostics, drug delivery, and regenerative applications.

### 3.1 Hydrophobic Interactions

**FIGURE 7:**
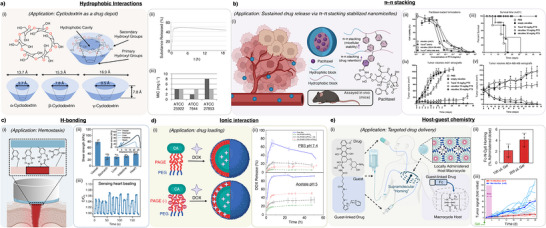
Spontaneous non‐covalent physical interactions and representative biomedical applications. (a). Graphical presentation of chemical structures and the molecular shape of cyclodextrins, whose hydrophobic cavities enable their feature to serve as drug delivery system (i). β‐Cyclodextrin complexation of meropenem achieved sustained release (ii) and reduced minimum inhibitory concentration (MIC) to *P. aeruginosa* ATCC 27853 (iii). Adapted with permission. [264, 267] Copyrights 2018, Wiley Periodicals, Inc.; 2015, Elsevier B.V. All rights reserved. (b) Targeted delivery and sustained release via dual‐block nanomicelles stabilized by *π*–*π* stacking (i). This delivery of paclitaxel decreased the half‐maximal inhibitory concentration (ii), enhanced survival in A431 cancer animal models (ii), and significantly inhibited the tumor volume of A431 xenografts (iv) and MDA‐MB‐488 xenografts (v). Adapted with permission. [293] Copyright 2015, American Chemical Society. (c) Enhanced adhesion and fault‐tolerant mechanism of Electro‐Ox hydrogel tape via H‐bonding and covalent bondings (i). The tape showed strong shear‐stress tolerance (ii), and enabled attachment of a strain sensor to a beating heart for pulse monitoring (iii). Scheme simplified based on the original concept and diagrams reused with permission. [296] Copyright 2021 The authors, distributed under the CC BY 4.0 license. (d). Ionic interactions enabling enhanced encapsulation (i) and sustained release (ii) of hydrophilic drug, exemplified by doxorubicin (DOX). CA, cholic acid. PAGE, poly(allyl glycidyl ether). PEG, poly(ethylene glycol). Scheme redrawn and diagrams reused with permission. [301] Copyright 2018, American Chemical Society. (e). Host‐guest chemistry applied for a “Homing” targeted delivery of guest‐linked drug to macrocycle host (i) providing a up to 6% administered guest accomplished homing functionality (ii), and homing prodrug (Fc‐Hdz‐Dox) showed significantly enhanced tumor inhibition to non‐homing variant (Me‐Hdz‐Dox, (iii). Fc, ferrocene. Hdz, hydrazone. Adapted with permission. [314] *This is an unofficial adaptation of an article that appeared in an ACS publication. ACS has not endorsed the content of this adaptation or the context of its use. Copyright 2019, American Chemical Society, licensed under terms of this Standard ACS AuthorChoice/Editors’ Choice usage agreement*.

Hydrophobic interactions refer to the spontaneous aggregation of amphiphilic or hydrophobic species in water to minimize solvent‐exposed nonpolar surface, without covalent bond formation [261, 262, 263]. The interacting species usually include host or carrier molecules bearing hydrophobic domains, such as cyclodextrins, amphiphilic block copolymers, and phospholipids, together with hydrophobic or amphiphilic cargo. The resulting products are non‐covalent supramolecular assemblies, including cyclodextrin complexes, micelles, and vesicles/liposomes. These assemblies are stabilized mainly by the hydrophobic effect with additional contributions from van der Waals interactions and hydrogen bonding. Representative categories and design variables include the following. (i) Cyclodextrins (α/β/γ‐CD) are cyclic oligosaccharides that encapsulates suitably sized hydrophobic guests within an apolar interior cavity while presenting a hydrophilic exterior. This improves apparent solubility and shelf stability (Figure 7a). Release kinetics are governed by equilibrium and are sensitive to ring size, substituents such as hydroxypropylation, and competitive displacement in vivo [264, 265, 266, 267, 268]. A representative example is β‐CD complexation of meropenem reported by Paczkowska et al. [267] In this study, hydrophobic interactions improved drug stability, sustained release for up to 20 h, and lowered the minimal inhibitory concentration (MIC) against *P. aeruginosa* (ATCC 27853) from 8.0 to 4.0 mg L^−1^. (ii) Amphiphilic block‐copolymer micelles self‐assemble into structures with hydrophobic cores that solubilize lipophilic drugs. This improves dispersion and passive delivery [269, 270], and the self‐assembly payload can be estimated using machine learning applications [271]. (iii) Liposomes hold hydrophilic cargoes in the aqueous core and lodge hydrophobic cargoes in the bilayer. This enables prolonged circulation and tunable release. However, they also demand formulation safeguards, such as cholesterol for membrane rigidity, PEGylation to limit opsonization, leakage and immune clearance [272, 273]. (iv) Albumin–fatty acid binding uses hydrophobic association between long‐chain fatty‐acid motifs and serum albumin to extend the circulation half‐life of peptides and proteins that would otherwise undergo rapid renal clearance [274]. Representative examples include insulin detemir [275], liraglutide [276], and semaglutide [277]. In these systems, fatty‐acid acylation promotes reversible albumin binding and thereby prolongs systemic exposure and dosing interval.

### 3.2 *π*–*π* Stacking and Hydrogen Bonding

*π*–*π* stacking and hydrogen bonding (H‐bonding) are directional, non‐covalent interactions that contribute extensively to molecular recognition and structural organization in living systems [278, 279, 280]. *π*–*π* stacking arise between aromatic *π*‐systems and is driven by dispersion and quadrupole‐driven interactions. H‐bonding requires donor‐acceptor pairs, such as amide, hydroxyl, carbonyl groups. Molecules bearing these motifs can spontaneously associate into reversible supramolecular complexes or higher‐order assemblies such as nanofibers, NPs, and hydrogels [279, 281, 282, 283, 284, 285]. These cooperative forces operate pervasively in physiology, including protein–ligand docking, receptor recognition, and nucleic‐acid stabilization [286, 287].

In biomedicine, *π*–*π* stacking and H‐bonding are increasingly used to guide supramolecular assembly. Carrier‐free small‐molecule nanodrugs, oligopeptides, and hydrogels can co‐assemble via cooperative *π*–*π* stacking and H‐bonding. This can produce high‐payload nanocarriers with passive tumor delivery and stimuli‐responsive disassembly [282, 288, 289, 290, 291]. The nanocarriers formed by hydrophobic assembly are also often stabilized or functionalized to harness *π*–*π* stacking and H‐bonding for targeted drug delivery (Figure 7b) [269, 292, 293, 294]. A representative example is the paclitaxel‐loaded polymeric micelle system reported by Shi et al. [293].

*π*–*π* stacking and H‐bonding were also incorporated into tissue‐interfacing biomaterials [295, 296, 297]. To exemplify, Xue et al. [296] developed catechol‐based hydrogel tapes that combined rapid physical adhesion with slower covalent fixation (Figure 7c). The tapes showed short‐term adhesion strengths above 30 kPa on wet tissues within <10 s. After 24 h, and long‐term adhesion strengths above 100 kPa, enabling stable attachment of a strain sensor to the beating porcine heart tissue.

Collectively, these interactions synergize to stabilize assemblies and enable adaptive, catalyst‐free control of drug loading and release in physiological environments.

### 3.3 Ionic Interactions

Ionic interactions are long‐range electrostatic attractions that arise between oppositely charged functional groups under physiological ionic strength [298, 299, 300]. Typical pairs include protonated amines (─NH_3_^+^) and deprotonated carboxylates (‐COO^−^), phosphates, or sulfates. These forces promote the formation of ion pairs [51], polyelectrolyte complexes [301], and coacervates [302, 303], from charged biomolecules or polymers. This process is driven in part through counterion release and charge neutralization, contributing to the stabilization of protein structures, the formation of enzyme‐substrate complexes, and the assembly of biomolecular structures [259, 304].

In vitro, ion exchange chromatography uses these charged‐based interaction to separate proteins [298]. In vivo, polyelectrolyte complexes and coacervates encapsulate payloads and tune release via pH and ionic strength (Figure 7d) [301, 302, 303, 305]. A representative example is the bile‐acid‐based block‐copolymer system reported by Cunningham et al. [301]. In this system, electrostatic loading of doxorubicin outperformed hydrophobic loading. It increased drug loading content to 14 wt.% with 72% encapsulation efficiency. The ionically loaded formulation also showed pH‐responsive release, with 51.5% release at pH 5 vs. 16.3% at pH 7.4 after 48 h.

Ionic interactions are also useful in bioadhesive materials. These materials can adhere to mucosal tissues and thereby improve the retention and effectiveness of the administered drugs [298, 306, 307]. Ionic interactions also facilitate the binding of drugs to charged cellular components, enhancing their uptake and therapeutic efficacy [301]. In addition, polyelectrolyte complexes and coacervates can protect encapsulated drugs from enzymatic degradation and ensuring their sustained release at the target site [301, 305].

### 3.4 Host‐Guest Chemistry

Host‐guest chemistry refers to the spontaneous and reversible inclusion of a molecular “guest” within a cavity‐bearing “host” [308, 309]. As an integrative modality, it builds on the fundamental non‐covalent forces described above. These include hydrophobic confinement, directional hydrogen bonding, and electrostatic recognition. Together, these interactions enable highly selective supramolecular assembly [310]. Classic hosts include CDs, crown ethers, cucurbiturils (CBs), and calixarenes. These hosts bind complementary guests, such as charged motifs, aromatics, metal complexes, or drugs in aqueous milieu. The binding is typically specific and reversible, enabling supramolecular sensors, switches, and drug‐carrier systems [308, 311, 312, 313].

In drug delivery, host‐guest complexation between a circulating guest and a locally deployed host offers a modular route to site‐biased accumulation. For example, Zou et al. [314] used a CB[7]‐rich injectable hydrogel as a local supramolecular homing depot (Figure 7e). The host–guest affinity was on the order of 10^12^ m ^−1^. This enabled localization of approximately 4% of a systemically administered model small molecule within hours in mice. The same depot also supported long‐lasting retention and serial reloading at same site upon repeated dosing [314]. In the same study, a ferrocene (Fc) guest‐modified doxorubicin prodrug (Fc‐Hdz‐Dox) slowed tumor growth more effectively than a nonbinding control prodrug (Me‐Hdz‐Dox) in an orthotopic breast tumor model. Both formulations remained well tolerated. (Figure 7e).

Host–guest networks have been engineered to respond to physiological stimuli (e.g., pH, ROS) to control the payload release [314, 315, 316]. This targeted release mechanism ensures that the drugs are delivered precisely where needed, minimizing side effects and improving therapeutic outcomes. Host‐guest chemistry is also used in the development of responsive biomaterials that change their properties in response to biological signals, providing advanced therapeutic and diagnostic capabilities [317, 318, 319].

### 3.5 Limitations

While not the primary emphasis of this review, hydrophobic assembly, *π*–*π* stacking and H‐bonding, ionic interactions, and host–guest chemistry provide orthogonal, spontaneous means to stabilize, solubilize, and localize therapeutic agents and probes [259, 260, 264, 298, 308, 317, 318, 319]. Their biomedical use, however, is constrained by context‐dependent instability in physiological environments. Hydrophobically assembled nanocarriers can dissociate after systemic dilution when local concentrations fall below the critical micellizations concentration (CMC), leading to accelerated unimer exchange and premature payload leakage unless ultralow‐CMC designs are implemented [320, 321]. Likewise, the structural integrity of hydrogen bonded and *π*–*π* stacked networks is susceptible to compromise in water‐rich media due to the competitive solvation and endogenous bindings to biomolecules [322, 323]. Ionic complexes are similarly sensitive to ambient physiological salts (∼150 mm), where salt‐mediated charge screening can reduce electrostatic attraction and destabilize polyelectrolyte assemblies [324]. Host–guest systems frequently suffer from competitive binding, as abundant native biological metabolites (such as cholesterol or aromatic amino acids) can prematurely outcompete and displace the engineered therapeutic guests from macrocyclic cavities [325, 326, 327]. Accordingly, these non‐covalent strategies are often most effective when molecular affinity, formulation stability, and the surrounding biological environment are carefully matched to the intended application, rather than relied on for persistent structural integrity alone [265, 305, 315].

## 4 Spontaneous Non‐Catalyzed In Vivo Degradation Pathways

In addition to non‐catalyzed conjugation reactions and non‐covalent interactions that can be exploited for biomedical design, physiological environments also support several spontaneous non‐catalyzed degradation pathways [328, 329, 330]. These processes are not the primary focus of this review, because they mainly involve cleavage or damage‐associated reactions rather than deliberate conjugation or molecular assembly. However, they provide important chemical and clinical context for understanding molecular stability, biomolecular modification, and disease‐associated chemistry in vivo. Therefore, this section briefly summarizes representative degradation pathways, including hydrolysis, oxidation, carbamylation, deamidation and isomerization.

### 4.1 Hydrolysis

Hydrolysis is one of the most common degradation pathways in physiological environments [331, 332, 333]. Many biologically important hydrolytic pathways in vivo are normally accelerated by enzymes such as esterases and amidases. Even without enzymatic catalysis, however, hydrolytically labile functional groups can still undergo hydrolysis in aqueous environments [332, 333]. This process is especially relevant to ester bonds [334, 335, 336], which are often susceptible to background cleavage across physiological pH ranges.

**FIGURE 8:**
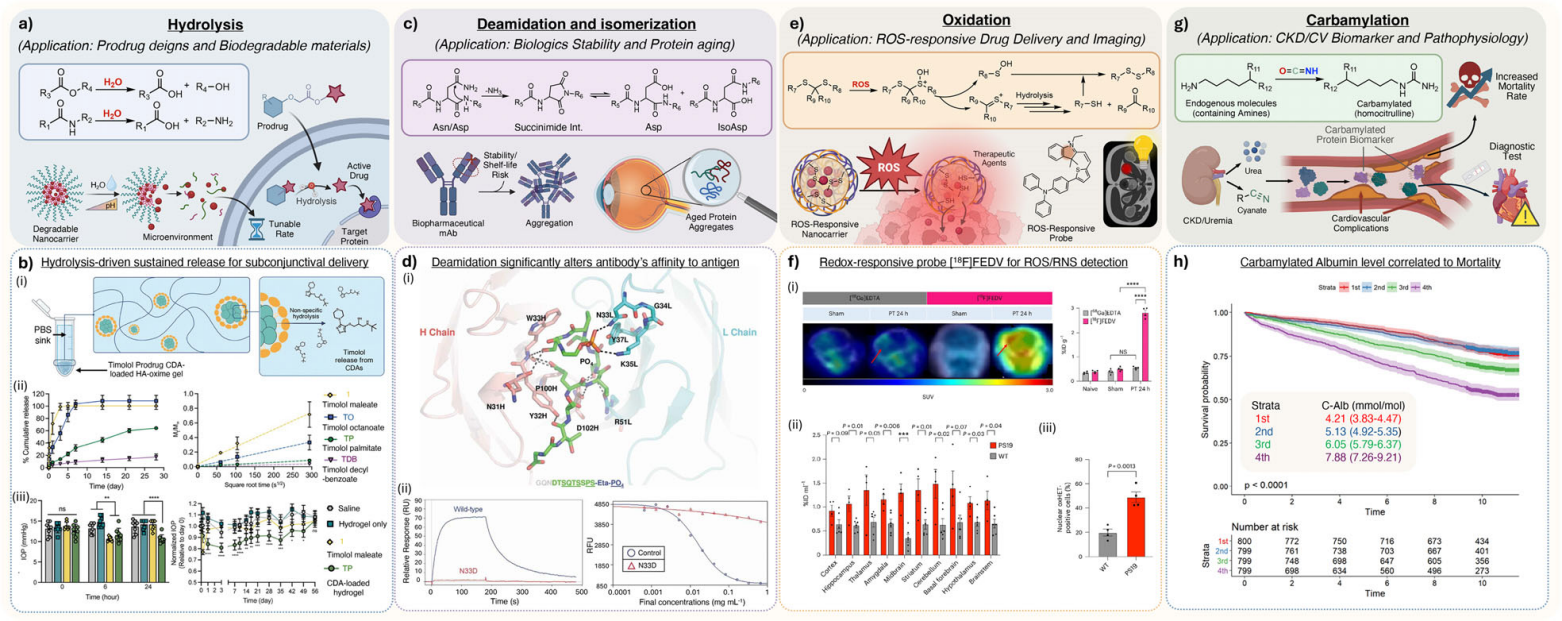
Representative spontaneous non‐catalyzed degradation pathways in vivo and their representative biomedical applications. (a). Hydrolysis of chemically labile groups (i.e., ester and amide bonds) utilized in prodrug designs [337] and biodegradable materials [338, 339]. (b). Hydrolysis‐driven sustained release from prodrug‐loaded hydrogels for subconjunctival delivery of timolol (i). The prodrug‐loaded hydrogel system achieved sustained release of timolol (ii) and significantly reduced intraocular pressure (IOP, iii). Reproduced with permission. [340] Copyright 2025, The Author(s). Advanced Materials published by Wiley‐VCH GmbH, distributed under CC‐BY‐NC‐ND license. (c). Succinimide‐mediated deamidation and isomerization applied in considerations for biologics stability [353, 354] and detection of protein aging [355, 356]. (d). Deamidation of Asn‐33 to an Asp (N33D) on the light chain of an anti‐CD52 antibody (MAB1, (i) markedly reduced its affinity for CD52. This was evidenced by significantly reduced responses in Biacore binding assay (ii) and complement‐dependent cytotoxicity assay (iii). CD, cluster of differentiation. RFU, relative fluorescent units. Reused with permission. [351] Copyright 2019, Taylor & Francis, distributed under CC‐BY‐NC‐ND license. (e). oxidation pathway adopted in reactive oxygen species (ROS)‐responsive drug delivery and imaging [362, 363, 371]. ROS‐responsive Probe structure adapted with permission. [361] Copyright 2025, Advanced Science published by Wiley‐VCH GmbH, distributed under CC BY License. (f). [^18^F]fluoroedaravone ([^18^F]FEDV), a redox‐responsive positron emission tomography (PET) probe which is ultrasensitive to ROS/RNS stress after photothrombotic (PT) injury (i). This probe also mapped ROS/RNS‐related oxidative stresses through the brain tissue in PS19 mice (ii) and quantified the oxidized hydroethidine (oxHET) positive nuclei (iii). Reused with permission. [368] Copyright 2025, The Author(s). Nature Biomedical Engineering published by Springer Nature, distributed under CC‐BY‐NC‐ND license. (g). Carbamylation in chronic kidney disease (CKD) [369] or cardiovascular (CV) complications [372] biomarkers and pathophysiology. (h). Association between carbamylated albumin levels and all‐cause mortality in patients with CKD. Reused with permission. [370] Copyright 2025, Association for Diagnostics & Laboratory Medicine 2025.

This predictable cleavage makes ester hydrolysis a commonly exploited mechanism for prodrugs [337] and degradable delivery systems [338, 339]. In these systems, programmed hydrolysis can regenerate the active parent drug, or trigger carrier disassembly after administration (Figure 8a). A representative example is the colloid‐forming timolol prodrug–hydrogel composite reported by Dang et al. [340]. In this design, hydrophobic timolol ester prodrugs were embedded in an HA‐oxime hydrogel and released by ester hydrolysis (Figure 8b). Timolol maleate was completely released within 48 h. In contrast, the prodrug colloid systems sustained timolol release for up to 28 days, with 37% and 82% of drug still retained in the TP‐ and TDB‐based hydrogels, respectively, after 28 days. After subconjunctival delivery of TP‐loaded hydrogel, the intraocular pressure (IOP) is significantly reduced and maintained in a reduced state through the entire test paradigm.

The rate of hydrolysis can often be modulated by steric properties, polarity, and the local microenvironment. In addition to ester bonds, amide bonds are also expected to hydrolyze even though the process is usually much slower under near‐neutral and enzyme‐free aqueous conditions [341, 342]. Their intrinsic stability is one reason biological systems generally rely on enzymes (e.g., amidases, proteases) for efficient proteolysis [343]. Therefore, hydrolysis is important both as a background source of molecular degradation in vivo and as a mechanistic basis for hydrolysis‐responsive biomedical design.

### 4.2 Deamidation and Isomerization

Deamidation and isomerization are important spontaneous pathways of protein instability in physiological environments. These processes are often mediated by cyclic succinimide intermediates. They involve interconversion among asparagine (Asn), aspartic acid (Asp), and isoaspartic acid (isoAsp). Briefly, Asn forms succinimide through deamidation [344, 345], whereas Asp forms succinimide through dehydration [346, 347, 348]. Subsequent hydrolysis of the cyclic succinimide intermediates generates mixtures of Asp and isoAsp residues (Figure 8c). These processes can alter local backbone connectivity, side‐chain arrangement, and higher‐order structure [349, 350], thereby affecting molecular recognition, conformational stability and protein function over time [351, 352].

These processes have attracted substantial attention in biopharmaceutical development [353, 354], where deamidation and isomerization are recognized as chemical liabilities that can compromise potency, stability, and shelf life. To exemplify, Qiu et al. [351] studied an anti‐CD52 antibody, MAB1, in which deamidation of Asn‐33 to Asp (N33D) in the light chain markedly reduced CD52 antigen binding (Figure 8d). In the Biacore binding assay, the deamidated variant showed an approximately 400‐fold loss in affinity. The functional consequence was further confirmed by reduced complement‐dependent cytotoxicity. The example shows how succinimide‐mediated Asn/Asp/isoAsp interconversion can directly compromise biotherapeutic potency. It also explains why motivate sequence engineering to remove such liabilities.

In addition, these processes have been implicated in age‐ and disease‐associated biomolecular damage. Examples include protein misfolding and aggregation, such as isoAsp‐linked aggregation in lens crystallins [352, 355, 356]. Together, these features make deamidation and isomerization important spontaneous pathways of protein instability in vivo. They are relevant both to therapeutic protein developability and age‐ and disease‐associated biomolecular damage.

### 4.3 Oxidation

Oxidation is an important degradation pathway in physiological environments. It is commonly driven by ROS and reactive nitrogen species (RNS). These species can modify susceptible amino acid residues, especially cysteine, methionine, and tyrosine, as well as functional groups such as disulfides and PBAs (Figure 8e) [7, 357, 358]. Depending on the oxidant, target and local microenvironment, oxidation can range from reversible modification to irreversible oxidative damage. These changes can alter molecular structure, activity, and stability.

In biological systems, it is closely associated with oxidative stress. It is often elevated in pathological settings such as inflammation, ischemia/reperfusion injury, and cancer. Because ROS/RNS levels are often higher in inflamed or tumor tissues than in many healthy tissues [359, 360], oxidation has also been well adopted as a trigger in biomedical designs. Representative applications include redox‐responsive fluorescent probes for imaging and diagnosis [360, 361], redox‐responsive prodrug [359] and drug‐delivery carriers [362, 363, 364] for pathologically selective activation or payload release, and smart biomaterials responsive to redox stimuli altering their structure, degradation or mechanical properties for regenerative medicines [365, 366, 367]. For example, Wilde et al. [368] reported a [^18^F] fluoroedaravone ([^18^F]FEDV) positron emission tomography (PET) probe (Figure 8f). In their design, oxidation by ROS and RNS generated a retained imaging signal for oxidative stress. In mouse stroke models, [^18^F]FEDV showed significantly greater uptake in the ipsilateral cortex at 24 h after photothrombotic (PT) injury, but not at 4 h. Dynamic positron emission tomography/magnetic resonance imaging (PET/MRI) further increased sensitivity, with significantly higher uptake rates in injured regions than in contralateral or sham controls. In 12‐month‐old PS19 mice, the probe also revealed widespread brain oxidative stress. This signal correlated with oxidized hydroethidine (oxHET) and phosphorylated tau (p‐tau) immunolabeling. Thus, oxidation in vivo is important not only as a source of biomolecular instabilities, but also useful triggers for diagnostics and therapeutics.

### 4.4 Carbamylation

Carbamylation is a spontaneous, non‐catalyzed modification reaction in which isocyanate or isocyanic acid reacts with protein amino groups, particularly Lys ε‐amines and N‐termini [329]. In vivo, this process is commonly associated with urea‐derived cyanate and can progress into more pronounced under conditions, such as uremia and chronic kidney disease (CKD, Figure 8g). [369] Carbamylation alters protein charge, structure, and function, thereby contributing to biomolecular dysfunction in pathological settings.

Owing to its clinical association with renal and inflammatory disorders, carbamylation has been widely studied as a biomarker‐related contexts. For example, Yazdiani et al. [370] measured carbamylated albumin (C‐Alb) in 3197 participants of the LURIC cohort and found the elevated C‐Alb was associated with increased all‐cause mortality (Figure 8h). The hazard ratio of 1.53 and 2.52 for the 3rd and 4th quartiles, respectively. The strongest association was observed for death due to congestive heart failure. In that case, the multivariate‐adjusted hazard ratio was 3.99 per 1‐unit increase in log‐transformed C‐Alb. Accordingly, carbamylation is important both as a spontaneous pathway of protein chemical damage in vivo and as a clinically relevant indicator of disease burden and a potential therapeutic target.

In summary, these degradation pathways demonstrate that spontaneous chemistry in vivo is not limited to useful conjugation reactions or non‐covalent interactions. It also includes background processes that often damage biomolecules, contribute to toxicity, or are regarded as chemical liabilities in biomedical systems. In selected contexts, some of these pathways can still be harnessed for controlled release, imaging, or disease monitoring. Although they are not the primary focus of this review, they provide important context for understanding spontaneous chemical behavior in physiological environments.

## 5 Outlook

Spontaneous, enzyme‐free chemistries and non‐covalent interactions offer physiologically compatible spatiotemporal control over therapeutic and diagnostic function in vivo. They can tune pharmacokinetics, enable covalent capture, or support reversible complexation under near‐neutral, aqueous conditions. These strategies are increasingly used across biomedical applications. Examples include dynamic glucose, catecholamine, ROS sensing; non‐catalyzed molecular tagging for imaging [39, 52, 87, 163, 230]; stimuli‐responsive carriers and prodrugs for controllable payload release [373, 374]; self‐healing dynamic materials for regeneration medicine [61, 124, 199]; and mucoadhesive, antifouling, immune‐modulated biointerfaces [295].

The selection of a non‐catalyzed strategy should be guided in part by its reaction or association timescale under physiological conditions (Table 3). (i) Ultrafast chemistries, which operate over seconds to minutes, are best suited to setting where reagents are dilute, transport‐limited, or in a time‐critical context. These conditions are common in live‐cell labeling, in vivo imaging, and rapid in situ ligation. Tetrazine‐TCO iEDDA is widely regarded as among the most instantaneous bioorthogonal ligations, with reported rate constants *k*_2_ = 10^3^–10^6^ m ^−1^ s^−1^ [48, 239, 240]. This makes it particularly attractive for pretargeted imaging and similar settings where short residence times demand rapid capture [233, 239, 241]. Thiol–Michael addition is also typically rapid (*k*_2_ = 10–10^4^ m ^−1^ s^−1^) [48, 203, 205, 206] and operationally simple with strong thiol preference around neutral pH. These features support fast site‐directed labeling and network formation. However, canonical maleimide thioethers may present instability in thiol‐rich biological milieus, necessitating stabilized designs when long‐term linkage integrity is required [207]. Boronate esterification is another fast and reversible covalent motif (*k*_2_ = 1–50 m ^−1^ s^−1^ via unfunctionalized PBAs [183, 184], 10^2^–10^3^ m ^−1^ s^−1^ via pK_a_‐engineered PBAs [185, 186, 187, 188]). Its dynamic association and dissociation kinetics make it highly effective for glucose sensing [162] and the creation of stimuli‐responsive depots [10, 375, 376]. At the same time, its strong dependence on pH and diol identity can make performance sensitive to complex biological microenvironments. Competing endogenous diols may interfere with binding and release behavior [10, 201, 377]. Non‐covalent interactions, including hydrophobic assembly, ionic complexation, and host–guest binding, also enable rapid self‐assembly of injectable biomaterials [378, 379, 380]. Despite this rapid association, the functional timescale of these nanocarriers is governed by their kinetic stability, with molecular architecture determining whether the assembly remains dynamic or becomes a rigid, non‐exchanging structure [381, 382]. In vivo, these assemblies can be challenged by complex biological microenvironments, where competitive salt ions and protein corona formation actively drive disassembly [383, 384].

(ii) Slower covalent chemistries, which often proceed over tens of minutes to hours, are generally better suited for sustained delivery, ex vivo conjugation, and material maturation. These settings allow pH, stoichiometry, and reaction time to be tightly managed rather than rapid imaging applications. NHS‐esters (*k*_2_ = 10^−1^ m ^−1^ s^−1^), for example, form highly stable amide bonds. However, this process often competes with aqueous hydrolysis and react broadly with primary amines, often yielding heterogeneous conjugates [385]. Imidoesters (apparent rate constant *k*’ = 0.08‐0.22 h^−1^ at pH 6.8–8.0) can efficiently modify amines while preserving cationic character, but they typically perform best under mildly basic conditions (≈ pH 8–10) [55]. Schiff bases and hydrazones are valuable for responsive biomaterials and sustained release. Their uncatalyzed kinetics near neutral pH are frequently slow (up to 2.85 m ^−1^ min^−1^ for Schiff base, ≤0.01 m ^−1^ s^−1^ for unmodified hydrazone) [42]. Catalysts, tuned buffers, or optimized substrates can accelerate rates substantially (up to ∼50 m ^−1^ s^−1^) [386, 387], but such strategies introduce additional constraints on biocompatibility, delivery, and local pH/buffer control.

(iii) Degradation or responsive pathways are well recognized as context‐dependent processes. Their kinetics are strongly influenced by substrate structure and local physiological environment. Hydrolysis is strongly bond dependent. Esters can undergo measurable and predictable background cleavage under physiological conditions [334, 335], whereas amide bonds hydrolyze much more slowly in physiological conditions [341, 342]. However, many hydrolytic processes are often ultrafast once with presence of corresponding enzymes in vivo [331, 332, 333]. Deamidation and isomerization are likewise sequence‐ and structure‐dependent spontaneous processes. Their rates depend on local residue environment, protein conformation, pH, and succinimide‐forming propensity [344, 345, 346, 347, 348]. Oxidation depends strongly on the local redox environment. Elevated ROS/RNS levels would significantly accelerate the cleavage processes, otherwise the redox‐responsive bonds are considered stable with ultraslow background cleavage [359, 360]. Carbamylation also lacks a fixed intrinsic rate across biological settings. Its extent depends on cyanate burden and therefore rises substantially in pathological environments such as uremia and CKD [329].

Collectively, this kinetics‐based framing highlights a central design principle. Ultrafast, highly selective ligations (e.g., iEDDA; thiol–Michael; boronate esterification) are favored for rapid labeling and imaging at low concentrations. Slower but robust couplings (e.g., NHS, imidoesters, imines/hydrazones) are better suited to ex vivo conjugation and time‐tolerant material maturation. Reversible covalent and supramolecular interactions (e.g., boronate esterification; hydrophobic, ionic, and host–guest assemblies) are preferred when stimuli‐responsiveness or self‐healing is desired. Their tradeoff is the greater sensitivity to biological microenvironments.

**FIGURE 9:**
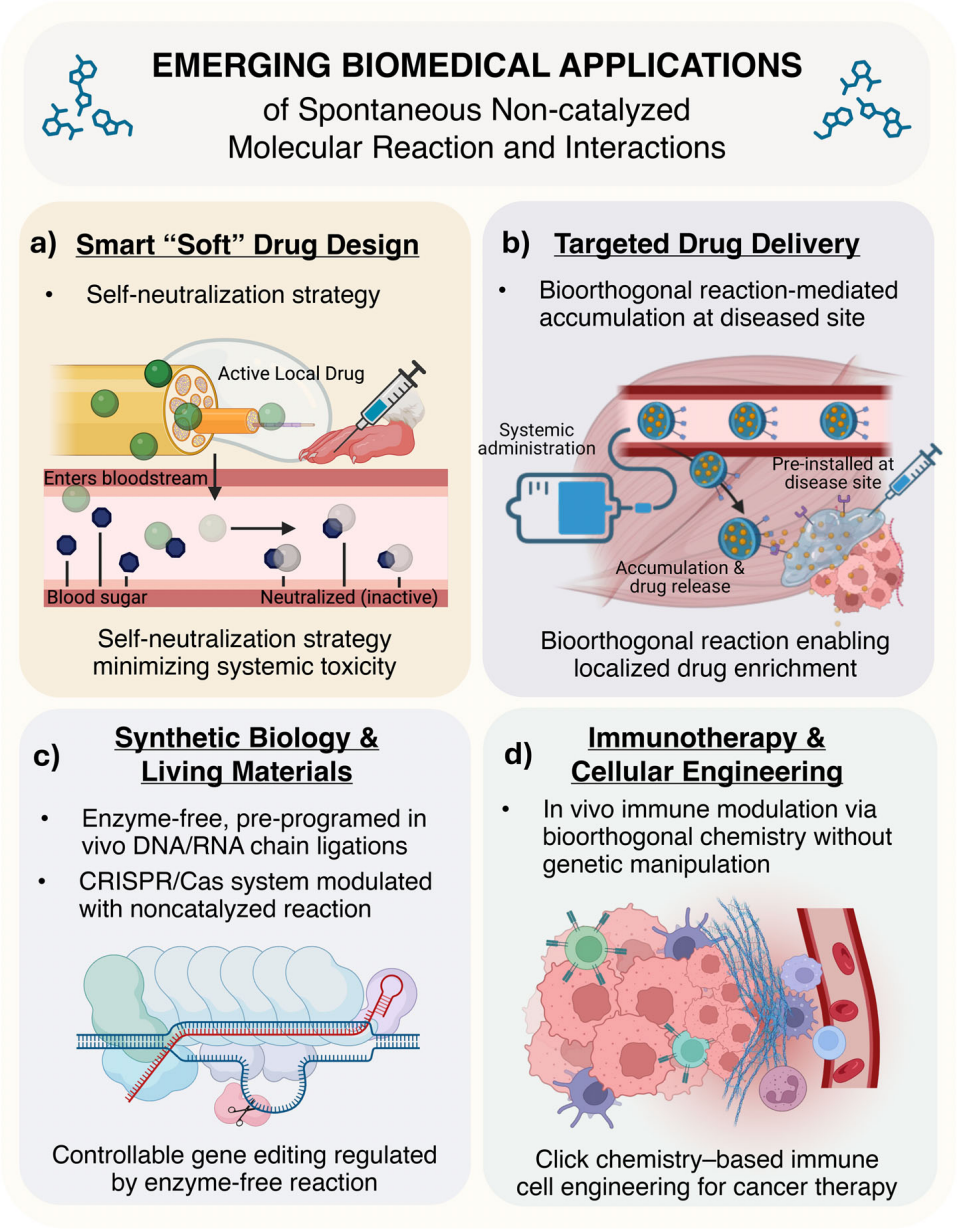
Outlook: emerging biomedical applications of spontaneous non‐catalyzed molecular reactions and interactions. (a) Self‐neutralizing “soft” drugs that retain pharmacological activity but are deactivated upon entering the systemic circulation by endogenous quenchers or physiological conditions. (b) Bioorthogonal reaction–mediated targeted drug delivery, in which systemically administered nanoparticles bearing complementary clickable groups are captured by a pre‐installed hydrogel depot near the diseased site and can trigger local drug release. (c) Programmable nucleic‐acid ligation for enzyme‐free control of genome‐editing systems (e.g., CRISPR‐Cas). Split guide‐RNA fragments bearing complementary dynamic‐covalent handles reconstitute a functional guide only when cognate parters meet. This can significantly contribute to synthetic biology; (d) in vivo, catalyst‐free immune‐cell functionalization for cancer immunotherapy, including bioorthogonal decoration of T‐cell surfaces and antibody‐guided capture of tagged immunomodulators at disease sites. All concepts operate under physiological conditions without added enzymes or metals. Schematics not to scale.

Beyond these established applications, we spotlight four emerging implications. First, smart “soft” drugs can be engineered with self‐neutralizing strategy (Figure 9a). These agents are designed to preserve pharmacological activity at the administration site. After entering systemic circulation, they are rapidly deactivated by endogenous quenchers or physiological conditions. This strategy offers a plausible route to reduce off‐target and systemic toxicity. Second, bioorthogonal reactions can support targeted delivery (Figure 9b). In one design, a hydrogel depot enriched with clickable handles is first injected or implanted near the diseased site. NPs bearing complementary bioorthogonal groups are then administered systemically. These NPs circulate and selectively react with depot. The click reaction captures the NPs near the target tissue and triggers local drug release. This approach increases on‐target exposure while reducing systemic burden. Third, programmable in vivo nucleic‐acid ligations may enable nonenzymatic control of genome‐editing systems (Figure 9c). For example, guide RNAs can be split into inactive fragments bearing complementary dynamic‐covalent handles, such as aldehyde/hydrazide pairs for hydrazone formation or boronic acid/diol pairs for boronate esterification. Only when the cognate partners meet do they ligate and reconstitute a functional full guide RNA. This restores ribonucleoprotein assembly and activates Cas9 or Cas12. Otherwise, the nuclease remains inactive. This affords stimuli‐responsive control of genome editing and advances minimal‐component synthetic biology [146]. However, its practical translation will depend on efficient co‐delivery of fragments to the same cells. It will also require suppression background ligation in serum‐like environments and sufficient stability of reactive handles during circulation [146, 388, 389]. Fourth, immunotherapy and cellular engineering can be implemented without genetic manipulation (Figure 9d). In situ, catalyst‐free bioorthogonal reactions can modify, functionalize, or activate immune cells directly in vivo. For example, iEDDA or SPAAC chemistries have been used to decorate T‐cell surfaces in vivo with cytokines, costimulatory ligands, or homing tags. Related click‐to‐release strategies can activate immune agonists at the immune synapse and boost cytotoxic function without ex vivo manipulation [249, 390]. In parallel, antibody‐guided modulation offers another route. In this strategy, a clickable tumor‐targeting antibody is first localized at the lesion. Tagged immunomodulators, such as IL‐2 variants or Toll‐like receptor agonists, are then captured in vivo at the disease site [12, 391]. This concentrates immune activity locally while limiting systemic exposure. These emerging strategies remain early in development, often at conceptional or proof‐of‐concept stages. Even so, they show strong applicational promise and are poised to substantially extended the biomedical impact of spontaneous, non‐catalyzed chemistry repertoire. With rational design, these processes can combine chemical controls with biocompatibility, establishing a foundation for autonomous, self‐regulating medical technologies.

Looking across mechanisms, a practical design framework is emerging. (i) Reaction kinetics should be matched to the in vivo residence time of reactive partners [12, 392]. (ii) Chemoselectivity must be modified against competing biomolecules, trigger sources, and biological stressors [393, 394, 395]. (iii) the chemistry should be coupled to mesoscale architecture, such as hydrogels, NPs, coatings or depots [13, 396, 397]. (iv) performance should be validated under realistic media, rather than only in idealized buffers [10, 398, 399]. For example, covalent handles such as NHS‐esters can enhance interfacial adhesion or durability in hydrogels and coatings. This benefit is most meaningful when the materials are tested in protein‐rich fluids and wet dynamic environments. Byproduct tolerance and off‐target amidation should be quantified. Such NHS‐based strategies have already improved tissue adhesion and stability in demanding biological settings [102]. Reversible diol binding via PBA motifs enables glucose‐responsive depots and adaptive materials. However, these systems require careful balancing of affinity, pH window, and competition from serum carbohydrates to maintain responsiveness in vivo [267]. Host–guest and ionic interactions can complement these covalent strategies. They can concentrate payloads, bias localization, and add failsafe release mechanisms under physiological stimuli such as pH and oxidative stresses [305, 316].

Spontaneous reactions can also be sequenced or spatially distributed to improve targeting across multiple sites. One example is a two‐patch microneedle (MN) system reported by Li et al. [400] In this design, one MN patch was made via NHS coupling, incorporating DBCO groups onto anti‐CD3/anti‐CD28 antibodies. This MN patch was applied over the lymph node region, thereby activating endogenous T cells and labeling them with DBCO groups. A second MN patch was applied at the tumor site and delivered N‐azidoacetylmannosamine‐tetraacylated (Ac4ManNAz) and IP10. Ac4ManNAz metabolically labeled tumor cell surface glycans with azide groups, while IP10 served as a T‐cell chemotactic factor. The resulting SPAAC reaction linked DBCO‐labeled T cells with azide‐labeled tumor cells, enhancing T‐cell infiltration and antitumor immunity in 4T1 tumor‐bearing mice. This example shows that spontaneous reactions can be spatially programmed to coordinate immune‐cell activation, migration, and tumor engagement in vivo.

Three near‐term priorities can accelerate translation. (i) Standardized benchmarks are needed. Studies should report second‐order rate constants and equilibrium parameters at pH 7.4 and 37°C in buffered saline. These measurements should also include serum challenge and selectivity panels against canonical amino acids and common metabolites [11¸ 12, 227, 248, 401, 402]. This would extend the fundamental in vitro rate constants summarized in Table 3 into more realistic biological settings. (ii) Materials testing should be mechanism‐aware. Molecular readout, such as conversion and exchange rate, should be linked with mechanical durability, including modulus, fatigue, and creep. Degradation should also be profiled under oxidative and enzymatic stress, so that network architecture is co‐designed with reaction reversibility [10, 216, 217, 373]. (iii) Safety and fate should be evaluated systemically. Important parameters include in vivo disposition of leaving groups and adducts, hemocompatibility, and immune activation. These issues are especially critical for high‐density conjugations and long‐lived depots. Hydrazine–carbonyl linkages are used as functional handles; it is still worth noting that hydrazine itself is not an endogenous primary amine. Its use therefore requires clear risk–benefit justification [248, 403, 404, 405].

In conclusion, spontaneous reactions and non‐covalent interactions in physiological environments provide a foundation for innovative solutions in healthcare. These processes can support selective capture, controlled release, adaptive materials, and disease‐responsive diagnostics. Their translation will require careful matching of reaction kinetics, selectivity, material architecture, and biological context. With this framework, spontaneous chemistry repertoire can help expand the range of safer, simpler, and more autonomous therapeutic and diagnostic technologies.

## Author Contributions

Y.C.: Writing – original draft, Investigation. C.Z: Writing – review & editing, Supervision, Methodology, Conception, Funding acquisition.

## Conflicts of Interest

The authors declare no conflicts of interest.
